# Bioinspired Oxygen‑Enriched Nanodiamonds as Electrolytic Erythrocyte Mimics for Dendrite‑Free Zinc‑Ion Batteries

**DOI:** 10.1002/advs.202518714

**Published:** 2025-11-21

**Authors:** Wenhao Ding, Wuxin Bai, Zhenjie Lu, Xiangjie Guo, Zhen Wu, Jingwen Sun, Pan Xiong, Wenyao Zhang, Xiaoping Ouyang, Xin Wang, Junwu Zhu, Yongsheng Fu

**Affiliations:** ^1^ Key Laboratory for Soft Chemistry and Functional Materials of Ministry of Education Nanjing University of Science and Technology Nanjing 210094 China; ^2^ School of Energy and Power Engineering Jiangsu University Zhenjiang 212013 China; ^3^ Key Laboratory of Low Dimensional Materials and Application Technology School of Materials Science and Engineering Xiangtan University Xiangtan 411105 China

**Keywords:** aqueous zinc‐ion batteries, charge‑reversible, dendrite suppression, high‑flux Zn^2+^ transport, oxygen‐enriched nanodiamonds

## Abstract

The implementation of aqueous zinc‑ion batteries (AZIBs) for large‑scale grid storage is thwarted by dendrite formation driven by concentration polarization. Drawing inspiration from the oxygen transport physiology of erythrocytes, oxygen‑enriched nanodiamonds (OND) are conceptualized as dynamic “electrolyte erythrocyte mimics” that enable intelligent Zn^2+^ shuttling under an applied electric field. These dynamic mediators operate through a reversible surface charge reversal mechanism: negatively charged OND initially adsorb Zn^2+^ at the anode, acquiring a positive zeta‑potential, then migrate cathode‐ward to release Zn^2+^ while regenerating their negative surface charge, completing a continuous transport cycle. Remarkably, demonstrates an exceptional transport capacity of 280 000 Zn^2+^ per cycle. In situ optical microscopy directly visualizes the OND‐driven Zn^2+^ trafficking between electrodes, resulting in exceptionally uniform and dense zinc deposition. Finite element simulations further reveal that OND induce beneficial micro‐convection in the electrolyte, simultaneously enhancing both zinc‐deposit homogeneity and electrolyte stability. Consequently, Zn//Zn symmetric cells sustain over 23 000 dendrite‐free cycles at 10 mA cm^−2^; Zn//Cu cells maintain 99.84 % Coulombic efficiency over 8600 cycles; and Zn//MnO_2_ full cells retain 89.1 % capacity after 10 000 cycles. This bioinspired ion shuttle strategy establishes a transformative approach for developing dendrite‑free metal batteries, unlocking the potential of AZIBs for safe, long‐duration grid storage.

## Graphical abstract

Inspired by the cyclic transport of oxygen from the lungs (high‑O_2_ region) to tissues (low‑O_2_ region), oxygen‑enriched nanodiamond (OND) is introduced into aqueous zinc‑ion batteries as “electrolyte erythrocytes.” These OND repeatedly shuttle Zn^2+^ from high‑ to low‑concentration regions, dramatically flattening the Zn^2+^ gradient and thereby effectively suppressing dendrite formation and parasitic reactions.

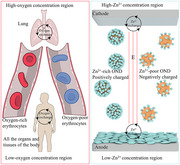

## 1 Introduction

Aqueous zinc‐ion batteries (AZIBs) have emerged as a highly promising candidate, offering an exceptional combination of high energy density, low cost, intrinsic safety, and environmental friendliness.^[^ ^1^, ^2^, ^3^, ^4^ ^]^ However, their practical deployment remains constrained by two intertwined issues: severe Zn^2+^ concentration polarization at the electrode‐electrolyte interface, and the resulting uncontrollable growth of zinc dendrites.^[^ ^5^, ^6^, ^7^ ^]^

This concentration polarization arises from sluggish Zn^2+^ transport kinetics during electrodeposition, which creates severe local ion depletion at the anode and cation accumulation at the cathode.^[^ ^8^, ^9^ ^]^ Such ionic inhomogeneity promotes uneven zinc nucleation and growth, ultimately forming dendrites.^[^ ^10^, ^11^ ^]^ These dendrites consume electrolyte and form electrically inactive “dead zinc,” and even penetrate the separator and cause short circuits, dramatically shortening cycle life. Previous studies have made significant progress in areas such as electrolyte formulations,^[^ ^12^, ^13^ ^]^ electrode interface modification^[^ ^14^, ^15^ ^]^ (such as artificial solid electrolyte interfaces and protective coatings), and the design of zinc anode micro/nano structures,^[^ ^16^ ^]^ effectively alleviating dendrite problems and enhancing cycle stability. However, ensuring a continuous and sufficient Zn^2+^ supply at the electrode–electrolyte interface to prevent concentration polarization remains a major challenge.

Studies have shown that enhancing cation flux can significantly mitigate concentration polarization.^[^ ^17^, ^18^ ^]^ As a simple yet efficient approach, electrolyte additives, owing to to their facile integration and cost‑effectiveness, have emerged as the preferred strategy for optimizing the ion transport at the AZIBs interface. For instance, carbon nitride quantum dots (≈10 nm) can host up to 185 Zn^2+^ and boost the transfer number of Zn^2+^ ($t_{Zn^{2+}}$) to 0.8, thereby markedly suppressing dendrite growth.^[^ ^19^ ^]^ However, these conventional high‐flux additives typically function unidirectionally within a single charge or discharge cycle and consequently accumulate near one electrode, quickly losing their regulatory function. In some cases, carrier accumulation may even cause localized clogging and induce new ionic inhomogeneities. In contrast, the oxygen transport physiology of erythrocytes provides an inspiring blueprint for dynamic ion shuttling: erythrocytes autonomously bind oxygen in the lungs (oxygen‐rich environment) and release it in oxygen‐deficient tissues, before returning to the lungs to repeat the process. Drawing inspiration from the oxygen transport physiology of erythrocytes, we conceptualize “electrolyte erythrocyte mimics” as additive that enable intelligent Zn^2+^ shuttling. In the context of AZIBs, such an additive should exhibit the following behavior: i) uptake of Zn^2+^ in high‐concentration regions, ii) migrate to low‐concentration regions, iii) release Zn^2+^ to facilitate uniform deposition, and iv) return to the high‐concentration region to reload Zn^2+^. To ensure continuous and efficient operation, these “electrolyte erythrocyte mimics” must simultaneously meet three critical criteria: 1) rapid and directional electrophoretic mobility under an electric field; 2) high Zn^2+^ loading capacity for ultrahigh flux; and 3) robust structural stability enabling fully reversible charge switching over thousands of cycles. Failure to satisfy any of these criteria would lead to precipitous performance decay.

**Figure 1:**
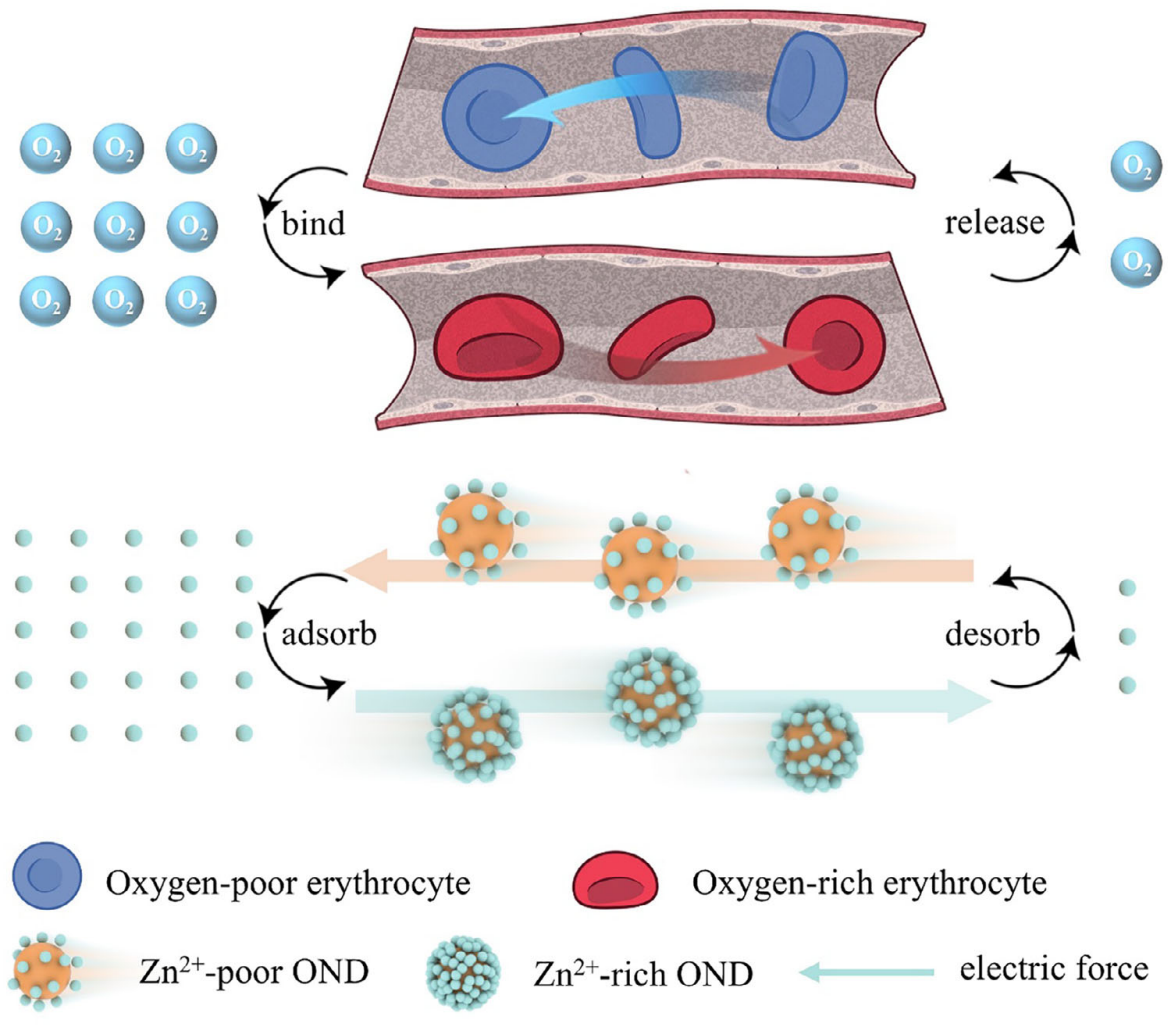
Schematic illustration of repeated O_2_ transport by erythrocytes and Zn^2+^ shuttling by OND. (Elements in this figure were adapted from NIAID NIH BioArt Source: NIAID Visual & Medical Arts. *Red Blood Cell*. bioart.niaid.nih.gov/bioart/442, /443, /444, NIAID Visual & Medical Arts. *Human Lungs*. bioart.niaid.nih.gov/bioart/231, NIAID Visual & Medical Arts. *Human Male Outline Type I*. bioart.niaid.nih.gov/bioart/233). NIAID Visual & Medical Arts. (10/7/2024). *Blood Vessel*. bioart.niaid.nih.gov/bioart/55 Public Domain, U.S. Government Work.

In this work, the oxygen‐enriched nanodiamonds (OND) are employed as “electrolytic erythrocyte mimics” to enable intelligent Zn^2+^ shuttling within electrolytes. The schematic diagram of the transport mechanism of OND is shown in **Figure** **1**. The erythrocytes bind O_2_ in oxygen‐rich regions, transport it through the vasculature to oxygen‐depleted tissues, and release O_2_, and this process repeats. Analogous to erythrocytes, OND act as dynamic Zn^2+^ shuttles. In Zn^2+^‑rich (high‑potential) regions, OND autonomously adsorb up to 280 000 Zn^2+^ per 300 nm particle, acquiring a positive zeta‑potential. Under an applied electric field, these Zn^2+^‑laden OND electrophorese into Zn^2+^‑depleted (low‑potential) zones, where they discharge their cargo and invert to a negative zeta‑potential, propelling them back to the Zn^2+^‑rich side. This continuous bidirectional cycle dramatically flattens interfacial concentration gradients, homogenizes ion distribution, suppresses dendrite growth, and sustains exceptional Coulombic efficiency. In situ optical microscopy vividly captures the repeated movement of OND and uniform zinc plating. Furthermore, the reciprocating motion of OND induces microscale electrolyte convection, which further enhances ion homogeneity, which is confirmed by COMSOL simulations. Consequently, Zn//Zn symmetric cells endure over 23 000 dendrite‑free cycles at 10 mA cm^−2^; Zn//Cu cells sustain 8600 cycles at 99.84 % average Coulombic efficiency; and Zn//MnO_2_ full cells retain 89.1 % capacity after 10 000 cycles. By merging dynamic surface chemistry with nanomaterial transport engineering, our OND shuttle strategy establishes a new guiding approach for stable, long‑life, grid‑scale AZIBs.

## 2 Results and Discussion

Nanodiamonds consist of a sp^3^‐hybridized diamond core sheathed in a sp^2^‐graphitized surface layer. Controlled surface graphitization followed by oxidative treatment yields oxygen‐enriched nanodiamonds (OND) bearing abundant oxygen‐containing functional groups. X‐ray photoelectron spectroscopy (XPS) confirms a pronounced increase in surface oxygen species and reveals a characteristic *π*–*π* ^*^ satellite feature from the graphitic shell (Figure S1, Supporting Information), with oxygen content rising from 8.7 % to 26.6 % (Table S1, Supporting Information). FTIR spectra further verify the presence of surface carboxyl and carbonyl groups (Figure S1, Supporting Information). X‐ray diffraction (XRD) patterns (Figure S1, Supporting Information) demonstrate preservation of the diamond core lattice, indicating surface‑confined oxidation. As shown in the Raman spectra (Figure S1, Supporting Information), the calculated I_D_/I_G_ ratio decreases from 2.55 for ND to 2.10 for OND, suggesting an increased degree of graphitization after the graphitization treatment and subsequent surface oxidation.^[^ ^20^ ^]^ Transmission electron microscopy (TEM) (Figure S2, Supporting Information) images reveal nearly spherical OND is ≈5 nm in diameter, which minimizes hydrodynamic drag in the electrolyte. High‐resolution TEM image displays lattice fringes corresponding to the (111) and (220) diamond planes. Elemental mapping confirms the uniform distribution of C and O throughout each OND.

The abundant surface functionality of OND is pivotal for its dispersibility and dynamic behavior. As shown in Figure S3 (Supporting Information), abundant carbonyl and carboxyl groups establish a robust electric double layer (EDL), yielding a zeta‑potential of −41 mV and ensuring excellent aqueous stability. Indeed, the OND dispersion remains uniform for over a month without visible sedimentation. Although introducing OND into a 2 m ZnSO_4_ electrolyte perturbs their EDL, charge neutralization does not trigger significant aggregation. The electrolyte containing OND showed no obvious precipitation after being left undisturbed for one week (Figure S4, Supporting Information). The average particle size of OND remained at ≈300 nm, which was much smaller than the pore diameter of the glass fiber (≈2.7 µm), thus ensuring that it could easily pass through the separator (Figure S5, Supporting Information). Moreover, OND increases the electrolyte viscosity only marginally due to the low additive content (1 wt.%) (Figure S6, Supporting Information). Correspondingly, the bulk ohmic resistance of a Zn//Zn symmetric cell rises slightly from 1.359  to 1.430 Ω upon OND addition (Figure S7, Supporting Information), with conductivity decreasing only from 43.9 to 41.2 mS cm^−1^, demonstrating that ionic transport remains essentially unaffected.

**Figure 2:**
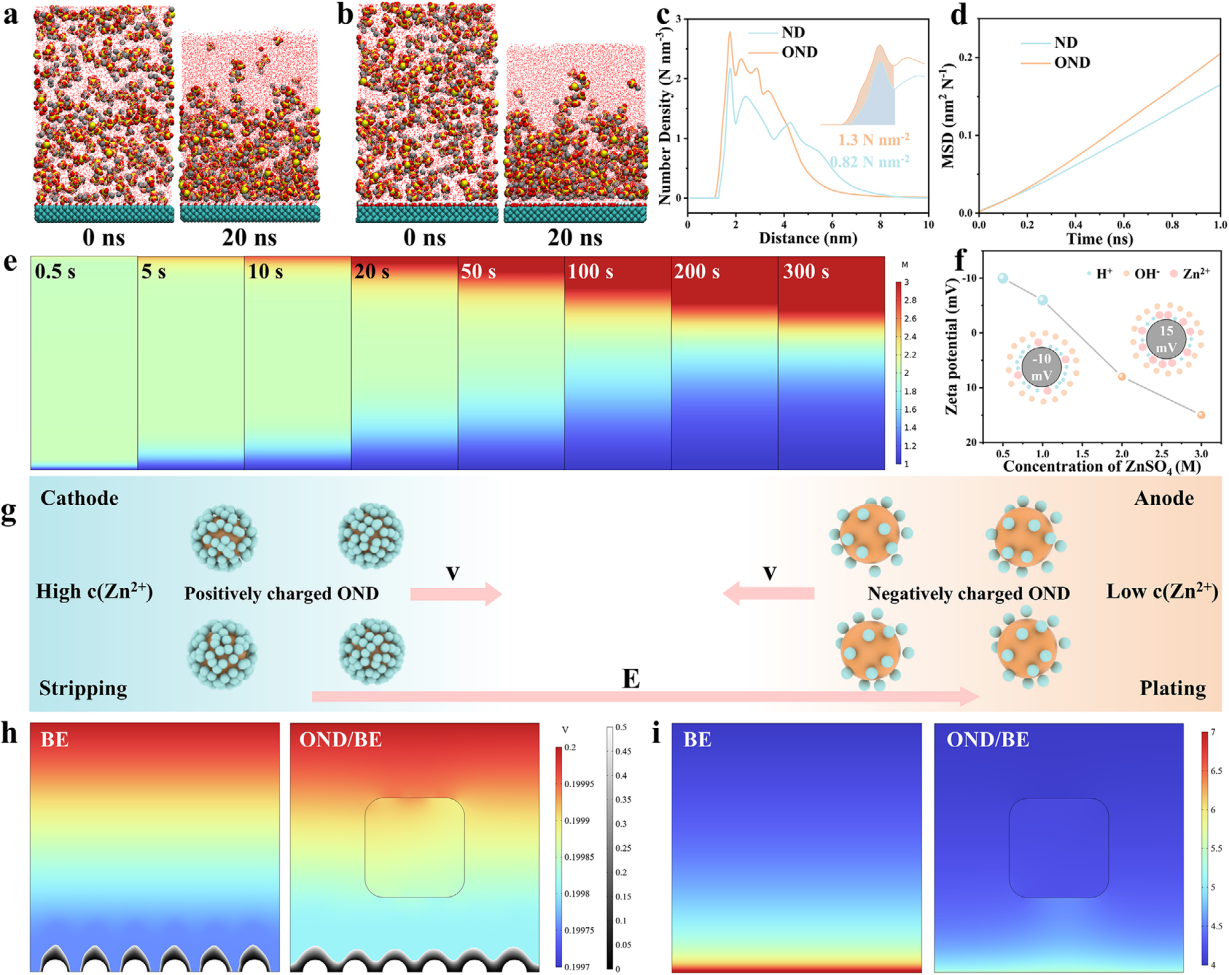
Molecular dynamics simulation of a) ND and b) OND immersed in ZnSO_4_ electrolyte; c) Number density profile of Zn^2+^ adjacent to ND and OND; d) Mean Squared Displacement (MSD) of Zn^2+^ on ND and OND; e) Spatiotemporal Zn^2+^ concentration maps in a Zn//Zn symmetric cell at 1 A g^−1^ over 0.5–300 s. f) Zeta potential of OND as a function of the concentration of ZnSO_4_, along with a schematic diagram of the change of the EDL of OND. g) Schematic of Zn^2+^ shuttling driven by continuous, charge‐reversal OND in the electrolyte. Finite element simulation: the micro‐disturbance effect of OND in the electrolyte on h) Zn deposition, i) pH distribution.

Molecular dynamics simulations were performed to reveal the microscopic mechanism of OND acting as dynamic “electrolytic erythrocytes.” After 20 ns of NPT equilibration, the results show that the Zn^2+^ adsorption density on OND with 26.6% oxygen content is markedly higher than that on OND with 8.7% oxygen (**Figure** **2**a,b). The higher oxygen content provides more abundant adsorption sites, granting the nanoparticles superior Zn^2+^‐loading capability. Integration of the first peak in the number density profile indicates that the surface density of adsorbed Zn^2+^ on OND reaches 1.3 N nm^−2^, compared to 0.82 N nm^−2^ on ND (Figure 2c). To further clarify the role of oxygenation, additional MD simulations were conducted for OND with oxygen contents of 8%, 17%, 26%, and 33%. As shown in Figure S8 (Supporting Information), the Zn^2+^ adsorption density increases monotonically with oxygen content, confirming a positive correlation between oxygen‐rich surface functionalization and Zn^2+^ affinity. Thus, a single 300 nm OND can theoretically adsorb ≈360 000 Zn^2+^. ICP measurements further confirm that a single OND adsorbs up to 280 000 Zn^2+^, consistent with the simulation results (Table S2, Supporting Information). Analysis of the MSD reveals that Zn^2+^ exhibits a higher diffusion coefficient in the high‐oxygen OND system, which facilitates faster ion adsorption (Figure 2d). Severe concentration polarization was also investigated using COMSOL simulations of Zn//Zn symmetric cells in 2 m ZnSO_4_ (Figure 2e). The Zn^2+^ concentration near the anode rapidly increases beyond 3 m, while that near the cathode drops below 1 m within 300 s. The zeta potential of OND varies with ZnSO_4_ concentration (Figure 2f). It is ≈−10 mV in 0.5 m ZnSO_4_, becomes neutral at ≈1.5 m, and turns positive at higher concentrations. This charge reversal arises because Zn^2+^ accumulates close to the OND surface at high concentrations, which partially compensates for the surface charge.^[^ ^21^, ^22^, ^23^, ^24^, ^25^ ^]^ This phenomenon forms the basis for the bidirectional shuttling behavior of OND (Figure 2g). In Zn^2+^‐rich regions, OND carry a positive surface charge and migrate toward the anode, transporting Zn^2+^. Upon reaching Zn^2+^‐depleted regions, they release the adsorbed Zn^2+^, regain a negative charge, and move back to the cathode. This cyclic motion continuously transports Zn^2+^ between concentration gradients, thereby alleviating polarization during operation. Moreover, the provision of high‐flux OND increased the migration number of Zn^2+^ from 0.52 in the blank electrolyte (BE, 2 m ZnSO_4_) to 0.73 in BE with OND (BE/OND) (Figure S9, Supporting Information).

The dynamic ion‐transport behavior of OND‐mediated electrolytes was probed by in situ optical microscopy (Figure S10, Supporting Information). Under static conditions, OND are uniformly dispersed throughout the electrolyte. Upon applying an electric field, the OND exhibits rapid, directional motion, continuously shuttling between anode and cathode in a cyclic manner (Video S1, Supporting Information). Therefore, rapid dendrite growth is observed in the BE at high current densities (10 mA cm^−2^), whereas the OND inclusion markedly significantly suppresses concentration polarization, yielding uniform and dense zinc deposition (Video S2, Supporting Information). Video observations reveal that the motion of OND induces microscale disturbances within the electrolyte, akin to gentle stirring, which enhance overall electrolyte homogeneity. As shown in Figure 2h,i, COMSOL finite element simulations further support this mechanism: microscale disturbances in the electrolyte cause a more uniform electric field distribution, resulting in smoother Zn deposition profiles and a more homogeneous interfacial pH gradient.

**Figure 3:**
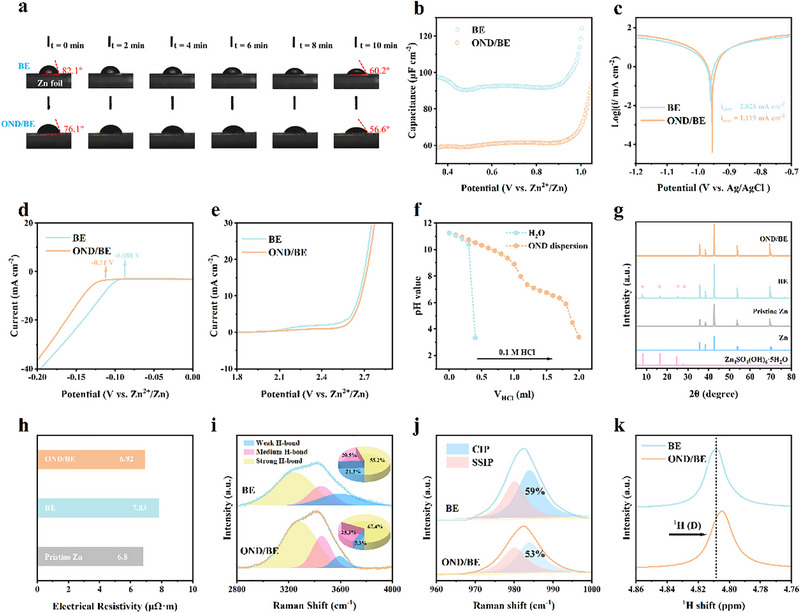
a) Contact angle of BE and OND/BE on Zn foil. b) Non‐Faradaic capacitance‐potential curves for the Cu electrodes. c) Linear polarization curve of the BE and OND/BE tested in a three‐electrode system. d,e) Linear voltammetry curves of Zn//Ti batteries at a scanning rate of 1 mV s^−1^. f) Titration performance of H_2_O and OND dispersions. g) XRD patterns and h) resistivity measurements of freshly polished Zn foils and those immersed in BE or OND/BE for three days (Zn_4_SO_4_(OH)_6_·5H_2_O PDF#39‐0688, Zn PDF#04‐0831). Raman spectra of BE and OND/BE at the Raman shift of i) 2800–4000 cm^−1^, j) 960–1000 cm^−1^. k) ^1^H NMR spectra of BE with/without OND additives.

In addition to providing high‐flux Zn^2+^ transport, OND plays multifunctional roles in stabilizing the anode. The carbonyl and carboxyl groups on OND, known for their high nucleophilicity, interact strongly with the surface of zinc, facilitating spontaneous adsorption and reinforcing the EDL.^[^ ^26^ ^]^ Consequently, the OND/BE exhibits improved wetting properties on the zinc (**Figure** **3**a).^[^ ^27^ ^]^ Differential capacitance curves (Figure 3b) show reduced capacitance due to OND displacing water and SO_4_^2−^, forming a thicker EDL.^[^ ^28^ ^]^ Thus, linear polarization curves (Figure 3c) demonstrate higher corrosion potential and reduced corrosion current. The electrochemical window of OND/BE is also broadened (Figure 3d,e), with the HER potential shifting from −0.088 to −0.11 V and the OER onset delayed. OND also exhibit notable pH buffering capabilities. As depicted in Figure 3f, the introduction of HCl into water results in a sharp decline in pH; while in the OND dispersion, it decreases more gradually. This behavior indicates that OND effectively moderates abrupt pH fluctuations, thereby aiding in the suppression of undesirable by‐product formation.^[^ ^28^ ^]^ After three days of immersion, BE‐treated zinc exhibited dendrites and sulfur by‐products, whereas OND‐protected zinc maintained a polished surface, with C elemental analysis confirming the adsorption of OND (Figure S11, Supporting Information). XRD (Figure 3g) showed by‐products only in BE‐immersed zinc, which also exhibited higher resistivity (7.83 µΩ·m), while OND‐protected zinc remained nearly constant (Figure 3h).

Deconvolution of the O─H stretching band (2800–4000 cm^−1^) reveals a higher fraction of strongly hydrogen‐bonded water in OND/BE compared to BE, indicating that OND modulate the hydrogen‐bonding network of the electrolyte (Figure 3i).^[^ ^29^, ^30^ ^]^ This structural rearrangement weakens the direct coordination between Zn^2+^ and H_2_O, thereby break and reorganize the hydrogen bond network. Moreover, the deconvolution of the sulfate band shows that OND reduce the proportion of contact ion pairs (CIP) while increasing solvent‐separated ion pairs (SSIP), confirming that OND inhibit the incorporation of SO_4_^2−^ into the Zn^2+^ primary solvation sheath (Figure 3j).^[^ ^31^, ^32^ ^]^ The intensity reduction of the [Zn(OH_2_)_6_]^2+^ feature (Figure S12, Supporting Information) further supports the disruption of the conventional hexahydrate coordination structure.^[^ ^19^ ^]^ In addition, the ^1^H NMR spectra (Figure 3k) exhibit an upfield chemical shift in D_2_O upon OND addition, implying weaker Zn^2+^‐H_2_O interaction and partial water release. This observation aligns with the blueshift of SO_4_^2−^ stretching in FTIR spectra (Figure S13, Supporting Information), collectively verifying a weakened solvation environment around Zn^2+^.^[^ ^33^ ^]^ Additionally, OND enhance electrolyte thermal management (Figure S14, Supporting Information), which can help expand the electrochemical window and improve cycling stability.^[^ ^34^ ^]^

**Figure 4:**
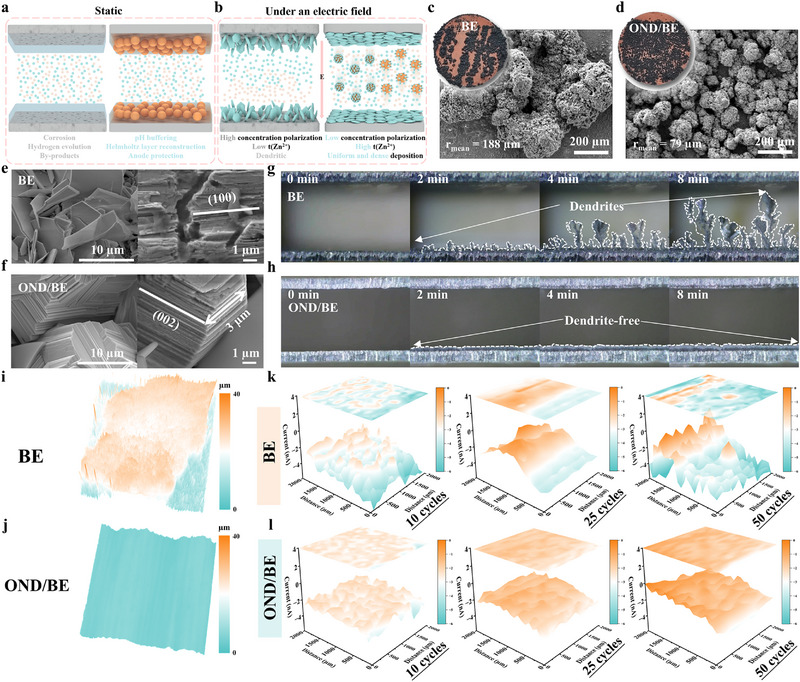
The diagram of OND improving the performance of zinc anode: a) at rest, b) under an applied electric field. Surface images of Cu foil after zinc deposition captured by a digital camera and an SEM image of the surface of Cu foil in c) BE, d) OND/BE. SEM images of the surface of Zn foil after 50 cycles in e) BE, f) OND/BE. Morphological changes during Zn^2+^ deposition at a current density of 10 mA cm^−2^ over different time intervals (0, 2, 4, 8 min) were observed using in situ optical microscopy in g) BE, h) OND/BE. LCSM images (2560 × 2560 µm) images of the surface of Zn foil after 50 cycles in i) BE, j) OND/BE. SECM images of the same zinc foil after 10, 25, and 50 cycles in k) BE, l) OND/BE.

During the resting stage, OND spontaneously adsorbs onto the surface of zinc, reconstructing the Helmholtz layer and effectively inhibiting undesirable reactions between H_2_O and zinc. Additionally, OND can rapidly neutralize OH^−^ in the electrolyte, thereby suppressing the formation of parasitic byproducts (**Figure** **4**a). Under an applied electric field, conventional electrolytes suffer from severe concentration polarization, which promotes dendrite growth, hydrogen evolution, and electrode corrosion. In contrast, OND enables high‐flux Zn^2+^ transport, significantly reducing polarization and promoting a more uniform electrolyte distribution. This dynamic regulation greatly suppresses dendrite formation, as illustrated in Figure 4b. To further understand the effect of OND on Zn^2+^ transport and interfacial deposition behavior, the desolvation energy of Zn^2+^ was quantitatively evaluated. As shown in Figure S15 (Supporting Information), the desolvation energy of Zn^2+^ in the BE is 57 kJ mol^−1^, whereas it increases to 61.2 kJ mol^−1^ in the OND/BE. This moderate increase suggests that the strong electrostatic interaction between the oxygen‐containing functional groups of OND and Zn^2+^ leads to the formation of a more stable solvation sheath. As shown in Figure S16 (Supporting Information), although OND participates in the solvation structure of Zn^2+^ and leads to an increase in the nucleation overpotential, this effect promotes denser zinc deposition.^[^ ^35^ ^]^ Zinc deposition was continuously carried out on the Cu foil (Figure 4c,d; Figure S17, Supporting Information), zinc particles in BE were larger and aggregated (188 µm radius), while OND/BE produced smaller, uniformly dispersed ones (79 µm). After 50 cycles, SEM images (Figure 4e,f) revealed spike‐like dendrites and loose deposition in BE, increasing surface area and “dead Zn” formation. In contrast, OND/BE formed compact deposits with (002)‐preferred orientation, enabling more stable growth. At a high current density of 10 mA cm^−2^, dendrites grow rapidly and extensively in BE, and their sharp tips can easily penetrate the separator, leading to cell failure. In contrast, with the presence of OND, Zn^2+^ concentration polarization is significantly reduced, enabling uniform and dense zinc deposition (Figure 4g,h). Laser confocal scanning microscopy (LCSM) reveals that the zinc cycled in BE exhibits a rough morphology with numerous sharp protrusions, whereas the zinc cycled in OND/BE is remarkably smooth and flat (Figure 4i,j). To assess morphological evolution and by‐product formation, scanning electrochemical microscopy (SECM) was conducted using a four‐electrode setup. The probe confirmed optimal performance through voltammetric testing (Figure S18, Supporting Information). As displayed in Figure 4k, dendrites and by‐products appeared after 10 cycles, became pronounced by 25 cycles, and severe by the 50th cycle. In comparison, Zn cycled in OND/BE remained smooth and compact up to 25 cycles, and even after 50 cycles, the current signal remained stable (Figure 4l), confirming uniform deposition and suppressed by‐product formation.^[^ ^36^ ^]^

**Figure 5:**
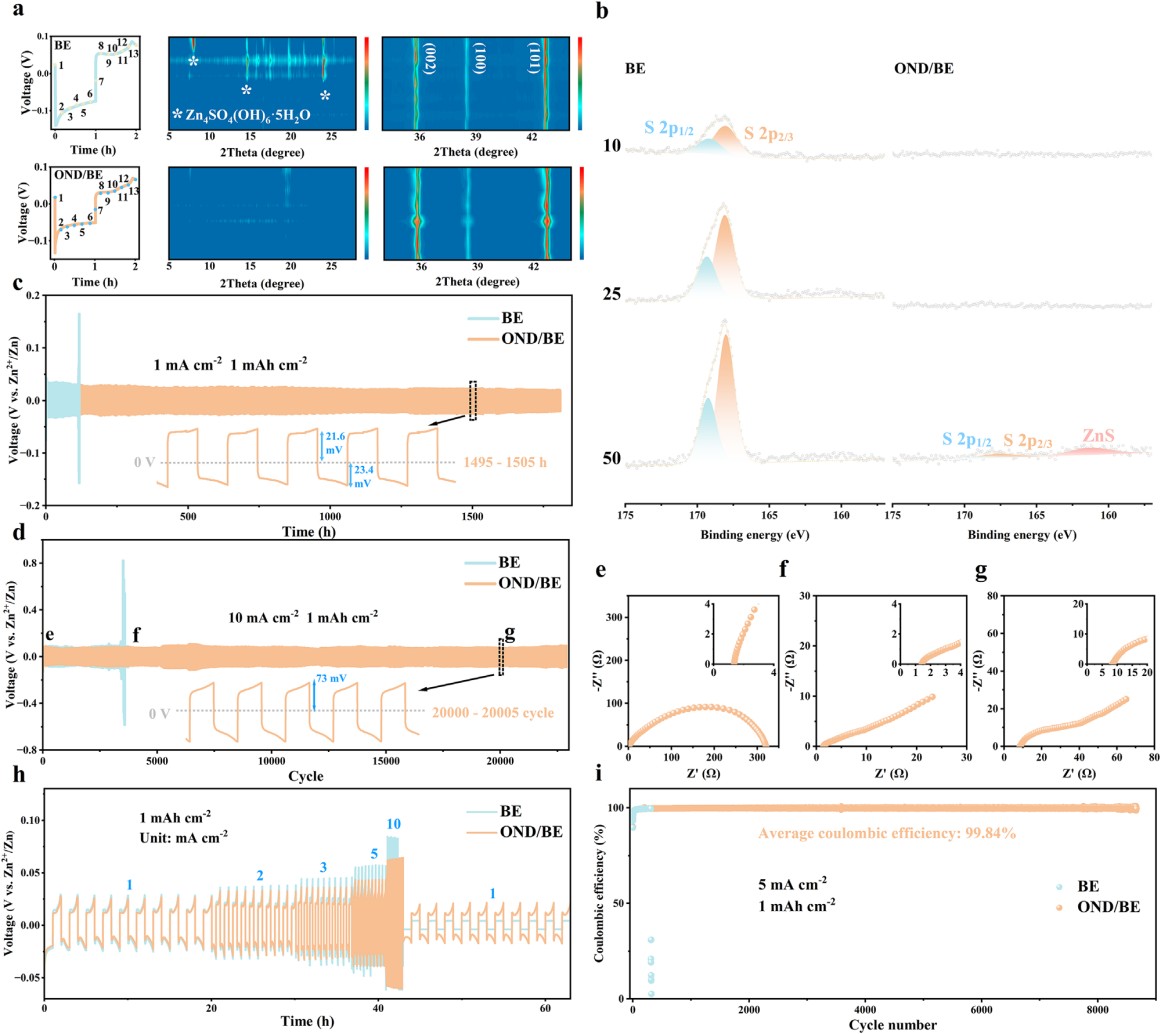
a) In situ XRD patterns of the positive electrode in Zn//Zn batteries at 1 mA cm^−2^ with different electrolytes. b) XPS spectra of Zn anodes after 10, 25, and 50 cycles in BE and OND/BE at 1 mA cm^−2^ and 1 mAh cm^−2^. c) Cycling performance of Zn//Zn cells at 1 mA cm^−2^ and 1 mAh cm^−2^. d) Long‐term galvanostatic Zn plating/stripping in Zn//Zn batteries under conditions of 10 mA cm^−2^ and 1 mAh cm^−2^. Impedance spectra of Zn//Zn cells in OND‐containing electrolyte after e) 400, f) 4000, g) 20 000 cycles. h) Rate performance of Zn//Zn batteries using BE and OND/BE. i) Coulombic efficiency of Zn//Cu cells at 5 mA cm^−2^ in different electrolytes.

To further investigate the influence of OND on Zn deposition and stripping behaviors, in situ XRD was performed on Zn//Zn symmetric batteries (**Figure** **5**a). During discharge, the Zn (002) peak at 35° significantly enhanced in OND/BE, indicating preferential deposition along the (002) plane. In contrast, peaks at 8°, 15°, and 24° during charging are observed, corresponding to Zn_4_SO_4_(OH)_6_·5H_2_O, a result of localized pH elevation.^[^ ^37^ ^]^ To further verify the suppression of by‐product formation, XPS was conducted on Zn anodes after 10, 25, and 50 cycles in BE and OND/BE. As shown in Figure 5b, clear S 2p signals appeared on the Zn surface cycled in BE electrolyte, corresponding to sulfate species in Zn_4_SO_4_(OH)_6_·5H_2_O. The intensity of the S 2p peak gradually increased with prolonged cycling, indicating the continuous accumulation of by‐products. In contrast, no obvious S 2p signal was observed for the Zn anodes cycled in OND/BE electrolyte at any stage, confirming that OND effectively suppresses the formation of Zn_4_SO_4_(OH)_6_·5H_2_O during repeated plating/stripping. In addition, SEM and elemental mapping analyses were performed on the Zn surfaces after 50 cycles (Figure S19, Supporting Information). The Zn anode cycled in BE electrolyte exhibited a rough and porous morphology covered with abundant nanosheet‐like deposits, which were identified as Zn_4_SO_4_(OH)_6_·5H_2_O by EDS, showing a strong sulfur signal. Conversely, the Zn anode cycled in OND/BE exhibited a smooth and compact surface morphology, with no sulfur detected in the corresponding mappings. These observations are consistent with the XRD and XPS results, collectively confirming that OND significantly inhibits side reactions and promotes uniform Zn deposition. The absence of these peaks in the OND/BE can be attributed to the dual functions of OND in suppressing hydrogen evolution and buffering pH.^[^ ^38^ ^]^ Batteries with BE experienced rapid polarization and short‐circuited after only 120 cycles, while OND‐containing cells achieved stable cycling for 1800 h (Figure 5c). Even at higher current densities (3 and 5 mA cm^−2^), OND‐enabled cells maintained stable operation for ≈5000 h (Figures S20 and S21, Supporting Information), with voltage hysteresis indicating stable battery performance, surpassing BE by factors of 18 and 12, respectively. Under extreme conditions (10 mA cm^−2^), OND‐containing batteries sustained long‐term cycling for up to 23 000 cycles s, while BE‐based cells failed after only 3500 cycles (Figure 5d). EIS tests after 400, 4000, and 20 000 cycles (Figure 5e–g) confirmed the absence of short‐circuiting or micro‐short‐circuiting in OND‐containing cells.^[^ ^35^, ^39^ ^]^

Rate performance tests (Figure 5h) revealed that cells with BE maintained reasonable performance at low current densities, they exhibited pronounced polarization and ultimately short‐circuited at 10 mA cm^−2^. In contrast, OND‐modified cells retained stable output with reduced polarization, attributed to OND‐enhanced Zn^2+^ transport kinetics. To evaluate Zn reversibility in different electrolytes, Zn//Cu asymmetric cells were assembled. At 5 mA cm^−2^, cells with BE failed after 300 cycles, while those with OND additives completed 8600 cycles and maintained an ultra‐high average coulomb efficiency of 99.84%, confirming the significant suppression of by‐products by OND (Figure 5i).^[^ ^40^ ^]^ Furthermore, as shown in Figure S22 (Supporting Information), OND enhanced Coulombic efficiency from the initial cycles and maintained lower voltage differentials, indicating minimal by‐product formation and improved internal stability. Zn//Ti cells were also employed to quantitatively assess Zn reversibility (Figure S23, Supporting Information). With BE, Coulombic efficiencies were 82%, 78%, 86%, and 87% in the 1st, 2nd, 5th, and 10th cycles, respectively. In contrast, OND increased these efficiencies to 86%, 81%, 89%, and 92%, demonstrating their ability to enhance Zn reversibility, reduce side reactions, and significantly improve battery stability and cycling life.^[^ ^41^, ^42^ ^]^ Therefore, the zinc symmetric battery of the OND‐based battery exhibits superior performance compared to the high‐performance electrolyte additives or coating strategies reported in the literature (Table S3, Supporting Information).

**Figure 6:**
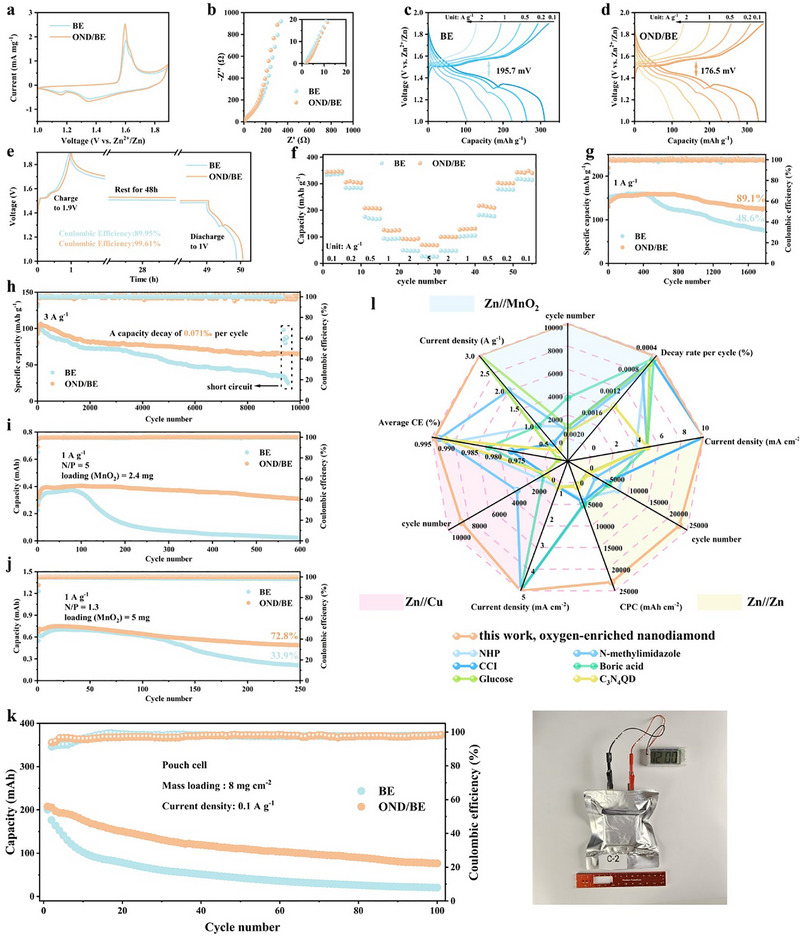
a) CV curves of Zn//MnO_2_ batteries with different electrolytes. b) EIS of the full cell. Charge–discharge performance at various current densities c) in BE, d) in OND/BE. e) Self‐discharge performance of the Zn//MnO_2_ cell. f) Rate performance of Zn//MnO_2_ batteries. g) Cycling stability of Zn//MnO_2_ cells at g) 1, h) 3 A g^−1^. Low N/P ratio battery cycle performance i) N/P = 5, j) N/P = 1.3. k) Cycling stability of pouch cells assembled with BE and OND/BE. l) A systematic comparison was conducted on the effects of high‐performance AZIBs electrolyte additives reported in the literature on the performance of Zn//Zn, Zn//Cu, and Zn//MnO_2_ batteries.^[^ ^19^, ^28^, ^33^, ^38^, ^43^, ^44^ ^]^

In our previous work, core–shell structured MnO_2_ was successfully synthesized and is employed in this study as the cathode material for Zn/MnO_2_ full batteries. Figures S24–S26 (Supporting Information) illustrate its core–shell structure, elemental distribution, and XRD spectrum, respectively. As shown in the CV curve (**Figure** **6**a), no significant changes were observed in the CV profile after the addition of OND, confirming the chemical stability of OND. Impedance spectra (Figure 6b) reveal a steeper low‐frequency slope for the OND‐based battery, indicating improved Zn^2+^ transport.^[^ ^45^ ^]^ Benefiting from the high‑flux Zn^2+^ transport mediated by OND, cells employing the OND/BE electrolyte exhibit markedly reduced polarization (Figure 6c,d). Self‐discharge tests (Figure 6e) reveal significantly higher Coulombic efficiency (≈100%) for OND‐based batteries compared to 89.95% for BE, confirming the suppression of side reactions. Moreover, the incorporation of OND increases the $t_{Zn^{2+}}$, enabling faster Zn^2+^ transport and thus delivering superior rate performance (Figure 6f). Cycling performance at 1 A g^−1^ (Figure 6g) reveals a sharp capacity decline in the BE‐based battery after 400 cycles, whereas the OND‐based battery retains 89.1% capacity after 1800 cycles. At 3 A g^−1^ (Figure 6h), the BE‐based battery experiences internal short‐circuiting after 9500 cycles, while the OND‐based battery maintains stable cycling over 10 000 cycles with an ultralow decay rate of 0.071‰ per cycle. To further assess the performance of OND under challenging conditions, batteries with low negative/positive (N/P) ratios were assembled. At N/P = 5 (Figure 6i), the BE‐based battery fails after 100 cycles due to side reactions and “dead zinc,” while the OND‐based battery retains stable capacity. At N/P = 1.3 (Figure 6j), the anode utilization rate reaches 77%. The battery employing BE retains only 33.4% capacity after 30 cycles, while OND maintains 72.8% capacity after 250 cycles. To further evaluate the practical feasibility of the OND‐modified electrolyte, pouch cells were assembled using BE and OND/BE electrolytes. As shown in Figure 6k, the cycling results reveal that the OND/BE‐based pouch cell exhibits markedly enhanced stability under high‐capacity conditions. After 100 cycles, the capacity retention is 36.8%, significantly higher than 10.3% for the BE‐based cell. These results unequivocally demonstrate the ability of OND to improve the reversibility and long‐term cycling performance of zinc‐based batteries. Thus, the cycle life and rate performance of the Zn//MnO_2_ battery based on OND are superior to those of most zinc‐based batteries that have been reported (Table S4, Supporting Information).

Figure 6l compares OND with previous aqueous electrolyte additives, demonstrating its superior performance across Zn//Zn, Zn//Cu, and Zn//MnO_2_ batteries. This dynamic movement strategy based on OND offers a significant advancement, delivering outstanding stability, reversibility, and cycling life, and holds great promise for the future of aqueous zinc‐ion batteries.

## 3 Conclusion

In this work, we have introduced OND as a bioinspired “electrolyte erythrocyte” that autonomously shuttles Zn^2+^ under an applied electric field, thereby overcoming the longstanding challenge of concentration polarization and dendrite growth in AZIBs. Through reversible surface‑charge reversal, each 300 nm OND carries an ultrahigh payload of 280 000 Zn^2+^ per cycle, adsorbing ions in high‑concentration zones and releasing them in depleted regions. In addition to enhancing the transport of Zn^2+^, OND inhibits parasitic reactions through three synergistic mechanisms: 1) reconstruction of the Helmholtz layer on the surface of the zinc negative electrode, 2) reconstruction of Zn^2+^ solvation structures, and 3) pH buffering via surface charge regulation. Remarkably, OND‐enabled zinc batteries achieve unprecedented cycling stability, sustaining over 23 000 cycles at 10 mA cm^−2^ in symmetric cells and maintaining 89.1% capacity retention after 10 000 cycles in Zn/MnO_2_ full cells. Even under extreme conditions (N/P = 1.3), the system retains 72.8% capacity after 250 cycles, demonstrating practical viability for high‐energy‐density applications. By marrying dynamic surface chemistry with nanoscale transport engineering, our OND shuttle strategy establishes a new paradigm for dendrite‑free metal anodes.

## Conflict of Interest

The authors declare no conflict of interest.

## Author Contributions

W.D. contributed to the writing of the original draft, methodology, formal analysis, and data curation. W.B. performed data curation. Z.L. conducted formal analysis. X.G. provided software; Z.W., J.S., and P.X. carried out investigation. W.Z. contributed methodology. X.O. performed formal analysis. X.W. provided resources. J.Z. oversaw the work, provided resources, and contributed to conceptualization. Y.F. contributed to writing—review and editing, supervision, project administration, formal analysis, and conceptualization.

## Supporting information



Supporting Information

Supplemental Video 1

Supplemental Video 2

## Data Availability

The data that support the findings of this study are available in the supplementary material of this article.
