# Supplementary material for: Bioinspired Oxygen‑Enriched Nanodiamonds as Electrolytic Erythrocyte Mimics for Dendrite‑Free Zinc‑Ion Batteries
Source: Adv Sci (Weinh). 2025 Nov 21;13(7):e18714. doi: 10.1002/advs.202518714 (PMC12866848; doi:10.1002/advs.202518714)
Supplement: Supplementary file 1 — Supporting Information [file ADVS-13-e18714-s002.docx]

**Supplementary Material**

**Bioinspired Oxygen‑Enriched Nanodiamonds as Electrolytic Erythrocyte Mimics for Dendrite‑Free Zinc‑Ion Batteries**

Wenhao Ding^a^, Wuxin Bai^a^, Zhenjie Lu^a^, Xiangjie Guo^a^, Zhen Wu^b^, Jingwen Sun^a^, Pan Xiong^a^, Wenyao Zhang^a^, Xiaoping Ouyang^c^, Xin Wang^a^, Junwu Zhu^a,*^, Yongsheng Fu^a,*^

*^a^* *Key Laboratory for Soft Chemistry and Functional Materials of Ministry of Education, Nanjing University of Science and Technology, Nanjing 210094, China*

*^b^* *School of Energy and Power Engineering, Jiangsu University, Zhenjiang 212013, China*

*^c^* *Key Laboratory of Low Dimensional Materials and Application Technology, School of Materials Science and Engineering, Xiangtan University, Xiangtan 411105, China.*

*Corresponding Author: zhujw@njust.edu.cn, fuyongsheng@njust.edu.cn

**Experimental Section**

**Materials.** ZnSO_4_·7H_2_O (AR, 99%), P_2_O_5_ (98%), Na_2_S_2_O_4_ (AR, 99%) was purchased from Aladdin, sulfuric acid (H_2_SO_4_), potassium permanganate (KMnO_4_), and hydrogen peroxide (H_2_O_2_) were purchased from the Chemical Reagent Center of Nanjing University of Science and Technology. Nanodiamonds (D50: 10 nm) were purchased from Nanjing Carry Foison Hard Materials Sci. & Tech. Co., Ltd., Zn foil (thickness of 100 μm), Cu foil (thickness of 10 μm) and Ti foil (thickness of 10 μm) were purchased from Shenzhen Kejing Star Technology. All other reagents were analytical grade and used directly without further purification.

**Materials Characterization.** The morphologies of the materials were examined using field-emission scanning electron microscopy (FESEM, JEOL7800F) in conjunction with energy dispersive X-ray spectroscopy (EDS) mapping. The TEM images were captured using the JEM-F200 instrument and analyzed for elemental composition using its built-in atomic-level mapping feature. Laser confocal microscopy was performed using Leica TCS SP8. The crystal structures were analyzed via X-ray powder diffraction utilizing Cu Kα radiation (λ = 0.15406 nm, MiniFlex600, Rigaku). Raman spectroscopy was conducted using a Renishaw inVia instrument with a 532 nm laser. NMR spectra were acquired employing a Bruker Avance III HD 500 MHz spectrometer from Bruker Co., Ltd., Switzerland. Contact angle measurements were performed using the OSA optical surface analyzer (OSA200, NBSI, China). The resistivity and conductivity of Zn foil were obtained using the RTS-11 metal four-probe testing instrument. The scanning electrochemical microscope images of Zn foil were obtained using Versa SCAN. The XPS spectra were obtained using the X-ray photoelectron spectroscopy (XPS) instrument (Thermo Scientific K-Alpha, America). The Zeta potential was measured using a Zeta potential analyzer (Malvern Zetasizer Nano ZS90, the UK). The viscosity was measured using a rheometer (Anton Paar MCR 302, Austria). The thermal conductivity of the electrolyte was measured using a thermal conductivity meter (Hot Disk TPS2500S, Sweden). The specific heat capacity was determined using the Differential Scanning Calorimetry sapphire method, employing the TA DSC2500 instrument from the America. The infrared spectra were obtained using a Fourier Transform Infrared (FTIR) spectrometer (Nicolet iS 10, America). The surface images of the Zn foil were obtained using a Laser Scanning Confocal Microscope (Leica TCS SP8). The pH value of the solution was measured using a pH meter (PHS-3C, LeiCi, Shanghai). The concentration of zinc ions was determined using inductively coupled plasma optical emission spectrometry (ICP-OES), with measurements performed on a PerkinElmer 8300 spectrometer.

**Preparation of oxygen-enriched nanodiamonds dispersion.** Oxygen-enriched nanodiamonds (OND) were prepared using the following method: First, detonation-synthesized nanodiamonds were heated in a tube furnace under an argon atmosphere at a heating rate of 10°C per minute to 1300°C and held for 4 hours to obtain surface-graphitized nanodiamonds. Then, 3 g of the surface-graphitized nanodiamonds were added to a mixture of concentrated sulfuric acid (120 mL), phosphorus pentoxide (P_2_O_5_, 0.3 g), and sodium dithionite (Na_2_S_2_O_4_, 1.2 g). The mixture was cooled to 0°C in an ice bath, and potassium permanganate (KMnO_4_, 9.0 g) was slowly added in portions while maintaining the reaction temperature below 4°C, followed by stirring for an additional 4 hours. After the reaction was completed, the temperature of the mixture was raised to 35°C and stirred for 3 hours. It was then transferred to room temperature and stirred vigorously for 5 days. Subsequently, the hot solution was poured into 800 mL of water (25°C), and 10 mL of 30% hydrogen peroxide (H_2_O_2_) was added to obtain a bright yellow solution. The solution was left to stand overnight, after which the supernatant was collected and placed in a dialysis bag. The water was changed every other day until neutrality was achieved, ultimately yielding an OND dispersion.

**Preparation of MnO_2_ cathodes.** Core-shell manganese dioxide is synthesized following a methodology outlined in a previous study.^[1]^ A solvent is prepared by mixing water and ethanol in a 4:1 ratio. Manganese dioxide, super p, LA133, and acrylic acid ester are combined in a proportion of 75:20:3:2. The mixture is then supplemented with an appropriate amount of solvent and ground to achieve a fine paste consistency. This paste is subsequently applied onto a titanium foil with a thickness of 10 µm. Afterward, the coated foil is subjected to drying at 120°C using forced air for 1 hour, followed by the cutting of circular discs with a diameter of 12 mm. The electrode demonstrates a loading capacity of approximately 1.5 mg cm^-2^.

**Battery Assembly.** ZnSO_4_·7H_2_O is added to the H_2_O to configure the concentration of 2M ZnSO_4_ solution, the modified electrolyte is to change the H_2_O into OND dispersion. All batteries are configured within CR2032 coin cells, with glass fiber (GF/D) serving as the separator and 100 µL of electrolyte. Cycling performance assessments of the coin cells are conducted utilizing a battery testing system (Neware battery tester, ShenZhen). For Zn//Zn symmetric cells employ Zn foil (100 µm) as the positive and negative electrodes, and various electrolytes are tested at current densities of 1, 3, 5 and 10 mA cm^-2^. Zn//Cu batteries feature a 10-µm Cu foil as the anode electrode and a 100-µm Zn foil as the cathode electrode. In the Zn//Ti battery, a 10-µm Ti foil serves as the anode, while a 100-µm Zn foil is employed as the cathode during assembly. In the Zn//MnO_2_ full cell, the positive electrode comprises a prepared MnO_2_ electrode, while the negative electrode consists of a 100-µm Zn foil. Cycling tests are performed at current densities of 1 A g^-1^ and 3 A g^-1^. A low N/P ratio Zn//MnO_2_ battery was prepared by preplating 1 mAh and 2 mAh Zn on a Cu net as the anode.

**Electrochemical test.** All electrochemical assessments are carried out using an electrochemical workstation (CHI 760E, Chenhua, Shanghai). Electrochemical impedance spectroscopy (EIS) is performed on Zn//Zn symmetrical cells across a frequency range spanning from 0.01 Hz to 100 kHz. To delineate the electrochemical windows of various electrolytes, Linear Sweep Voltammetry (LSV) experiments are conducted on the Zn//Ti battery employing a scan rate of 10 mV s^-1^. For electrochemical corrosion assessment, a three-electrode system is employed, with Zn acting as the working electrode, an Ag/AgCl electrode as the reference electrode, and a Pt electrode as the counter electrode. The voltage range for this test is set at ±0.3 V relative to the open circuit voltage, with a scan rate of 1 mV s^-1^. In the case of the Zn//Ti battery, cyclic voltammetry (CV) is performed within a voltage range of -0.2 V to 0.5 V at a scan rate of 1 mV s^-1^. For electrodeposition on the Zn//Zn symmetric cell, assembly is followed by subjecting it to a constant potential of -150 mV, yielding a current-against-time (CA) curve. The CV curve for the Zn-MnO_2_ full cell is generated within a voltage range spanning from 1 V to 1.9 V, with a scan rate of 1 mV s^-1^. Zn//MnO_2_ batteries have impedance frequencies ranging from 0.01 Hz to 100 kHz. The differential capacitance curve in BE and OND/BE is calculated using Equation $C={-(\omega Z_{im})}^{-1}$, where *C* is the differential capacitance, *ω* is the angular frequency, and *Z_im_* is the imaginary part of impedance, and 1000 Hz was selected as the specific frequency.

**Theoritical calculations.** Molecular dynamics (MD) simulation was performed by Gromacs2020.7 software.^[2]^ The boxes were built by filling molecules randomly using Packmol program. The force field parameters of Zn^2+^ and tip4p water model was got from OPLS-AA force field.^[3]^ RESP atom charges were used to describe electrostatic interactions. Atomic charges of Zn^2+^, SO_4_^2-^ were multiplied by scale factor 0.7 to correct the polarization effect of ions. The molecular force field is consisted of nonbonded and bonded interaction. The nonbonded interaction contains van deer Waals (vdW) and electrostatic interaction. For this simulation, an energy minimization was firstly employed to relax the simulation box. Then, an isothermal-isobaric (NPT) ensemble with a 1.0 fs time step is employed to optimized the simulation box, where the temperature is set to 298 K and the pressure is set to 1.0 atm. The temperature and pressure are kept via the Nose-Hoover thermostat and Parrinello-Rahman barostat, respectively. The NPT optimization time was set to 5.0 ns, which is enough long to obtain a stable box size. Following the NPT simulation, a canonical (NVT) ensemble with 10.0 ns was performed to furtherly optimize the simulation box, the time step is set to 2.0 fs. In all the MD simulation, the motion of atoms was described by classical Newton’s equation, which was solved using the velocity-Verlet algorithm.

Based on COMSOL Multiphysics 6.2, we established a numerical model where the zinc electrode was represented by a rectangular domain with a width of 125 μm and a height of 250 μm. The bulk concentration distribution of zinc ions was calculated using the Nernst-Planck equations. The simulation outputs were recorded at time intervals of range (0, 0.5, 300) s (i.e., from 0 to 300 s with a 0.5 s increment). In this system, the ambient temperature was set to 298.15 K (25°C), and the initial concentration of the electrolyte (ZnSO_4_ aqueous solution) was fixed at 2 M (mol·L^-1^).

Using COMSOL Multiphysics 6.2, a two-dimensional model was established to compare the effects of dynamic single-ion conductive electrolyte constructed by OND on the distribution of electric field, ion concentration, and zinc deposition during the circulation process. The finite element method was employed, utilizing a third-order current distribution module. A rotating domain is set in the middle to simulate the dynamic motion of the electrolyte driven by OND. The operating voltage was set to 0.2 V, with an exchange current density of 150 A m^-2^, and the initial concentration of Zn^2+^ was set to 2 mol L^-1^. Deposition time was 30 seconds. The motion of charged ions was described by the Nernst-Planck equation,

where represents the ion diffusion coefficient, is the ion charge number, is the temperature in Kelvin, is Faraday's constant, is the ideal gas constant, and is the potential.

The reaction on the electrode surface follows the Butler-Volmer equation,

where represents the electrode current density, is the exchange current density, is the anodic charge transfer coefficient, is the cathodic charge transfer coefficient, is Faraday's constant, is the overpotential, is the temperature in Kelvin, and is the ideal gas constant.

The rotating electrolyte model adds a rotating domain to the original model, and the internal fluid flow follows the Navier-Stokes equations,

Where represents fluid density, is the velocity vector, is the external force per unit volume acting on the fluid, is pressure.





**Figure S1.** Characterization of ND and OND. XPS spectra of (a) C1s, (b) O1s, and (c) survey spectra. (d) FTIR spectra, (e) XRD pattern, (f) Raman spectra.


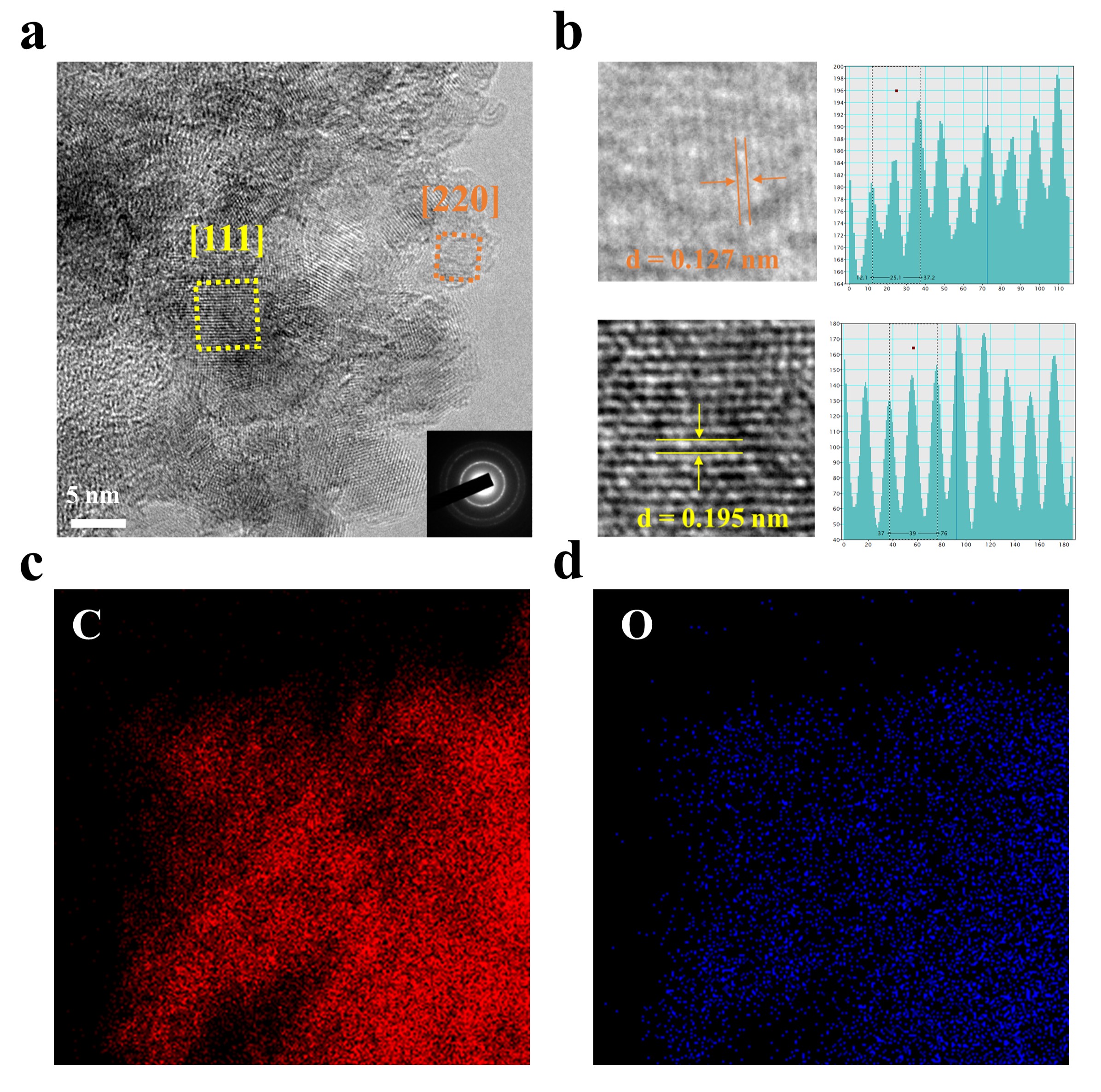


**Figure S2.** (a) High-resolution image (inset showing SAED pattern), (b) Measurement of the spacing of lattice stripes, elemental distribution of (c) C, (d) O.


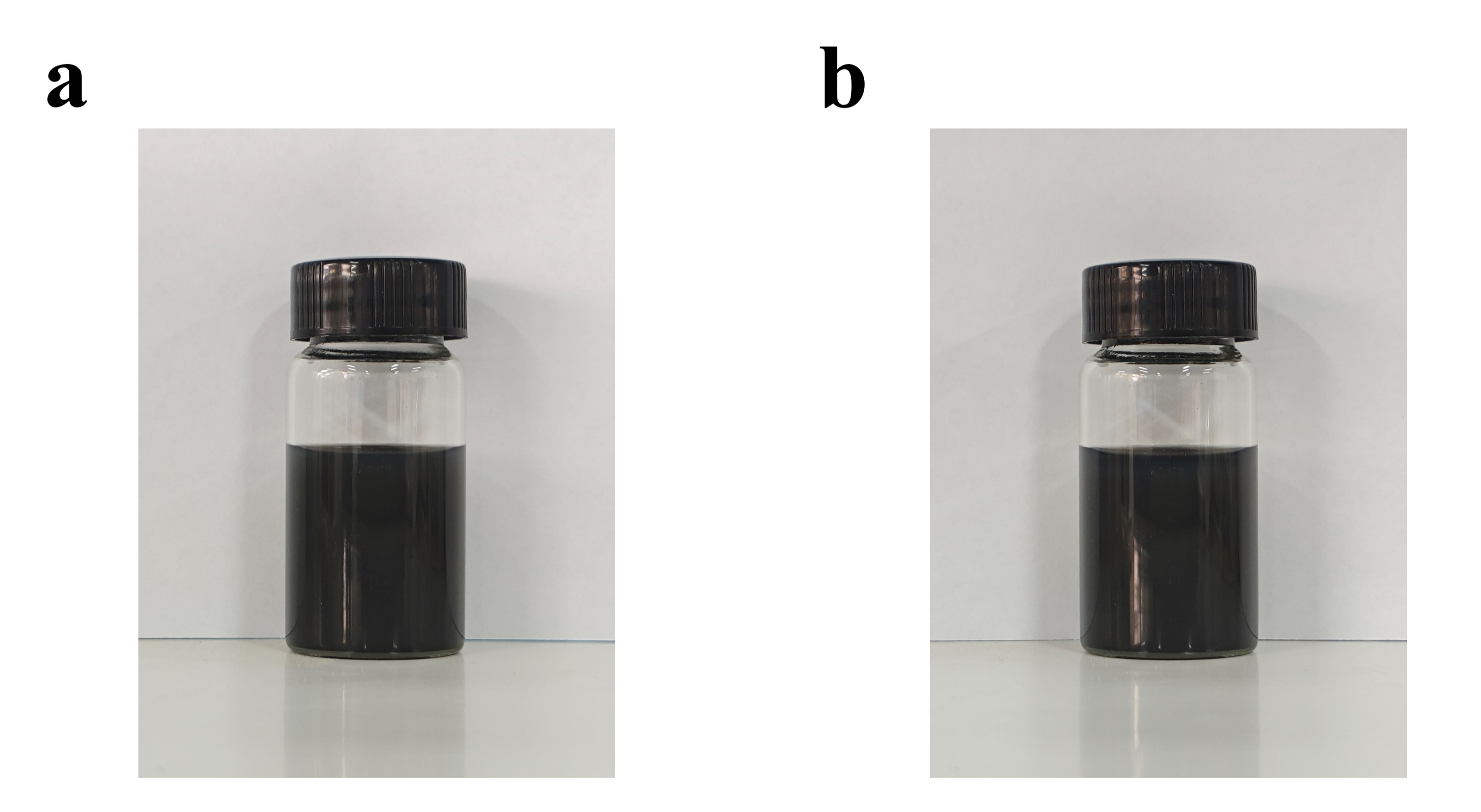


**Figure S3.** (a) OND dispersion, (b) OND dispersion after standing for one month.


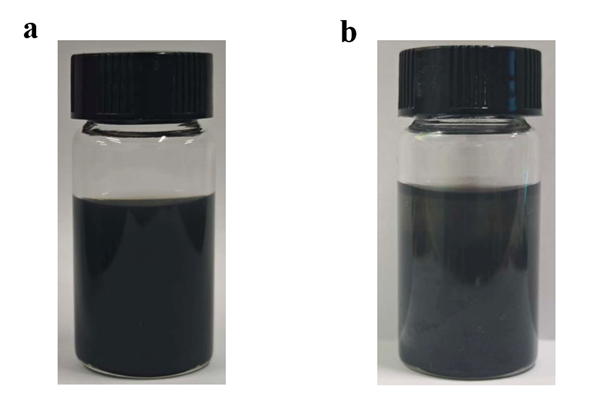


**Figure S4.** (a) OND in 2M ZnSO_4_, (b) OND in 2M ZnSO_4_ after standing for 3 days.





**Figure S5.** Particle size distribution of OND dispersion and OND in 2M ZnSO_4_.





**Figure S6.** Viscosity comparison of BE and BE/OND electrolytes.





**Figure S7.** Electrochemical impedance spectra (EIS) of Zn//Zn symmetric cells in different electrolytes.

The ionic conductivity was determined using the formula:

$$\sigma=\frac{L}{RA}$$

where *R* represents the series resistance, *L* denotes the thickness of the separator placed between the zinc electrodes (675 μm), and *A* stands for the total surface area of the titanium electrode (1.13 cm^2^).


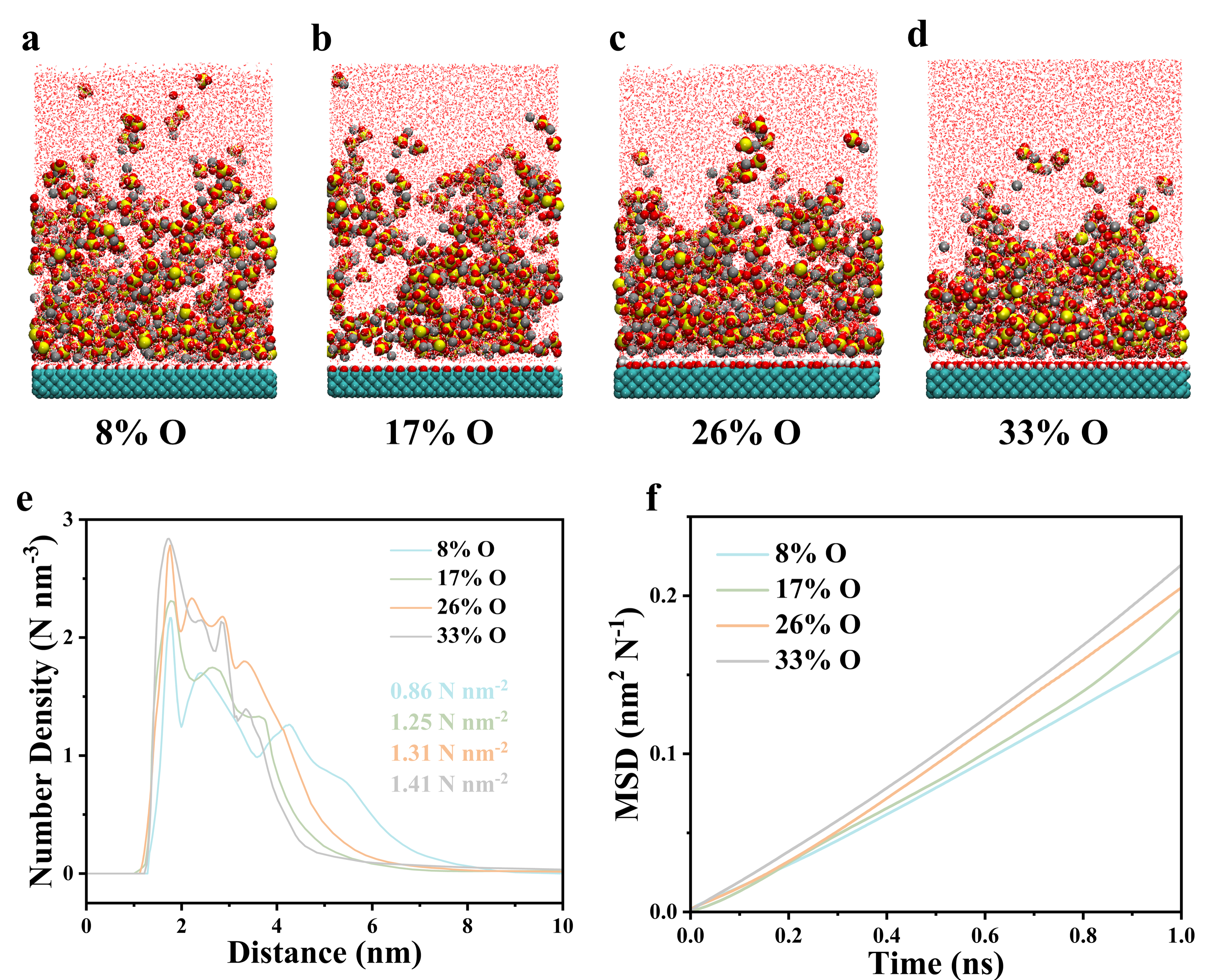


**Figure S8.** Representative snapshots of Zn^2+^ distribution near OND surfaces with different oxygen contents after 20 ns of MD equilibration (a) 8%, (b) 17%, (c) 26%, and (d) 33%. (e) Number density profiles of Zn^2+^ as a function of distance from the OND surface for different oxygen contents. (c) Mean square displacement (MSD) of Zn^2+^ near OND surfaces with varying oxygen contents.


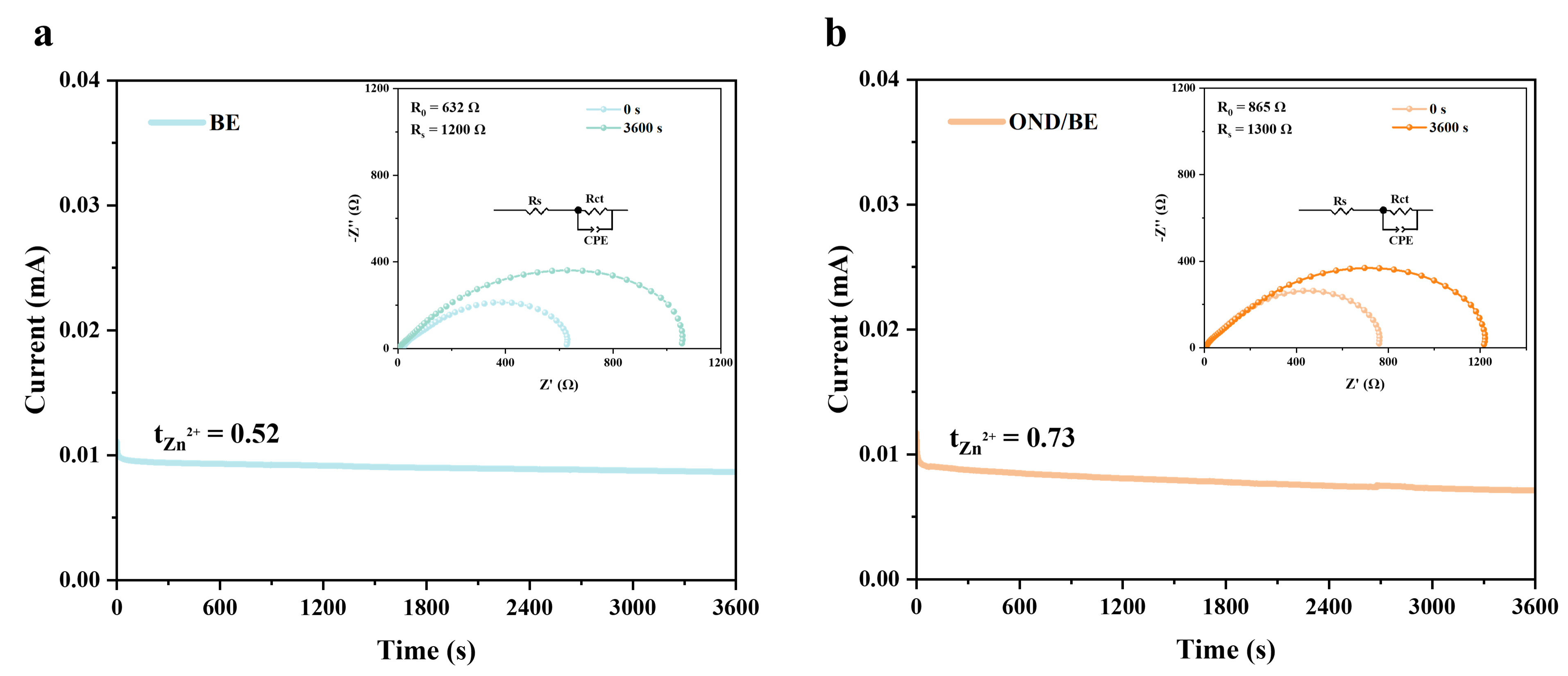


**Figure S9.** The transfer number of Zn^2+^ tested at an overpotential of 25 mV in (a) BE, (b) OND/BE.


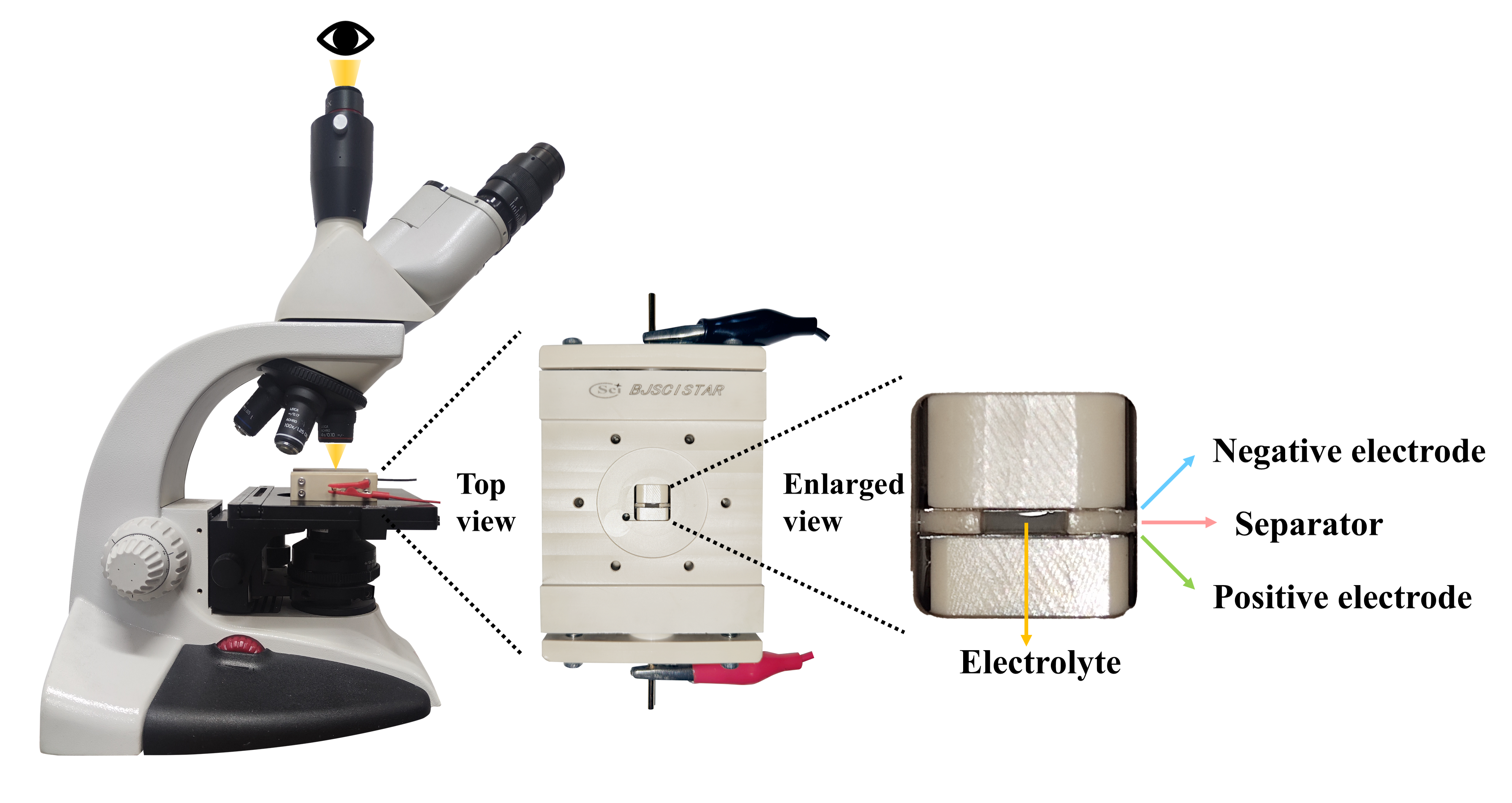


**Figure S10.** Diagram of in-situ optical microscope schematic, observing through the eyepiece and capturing video.


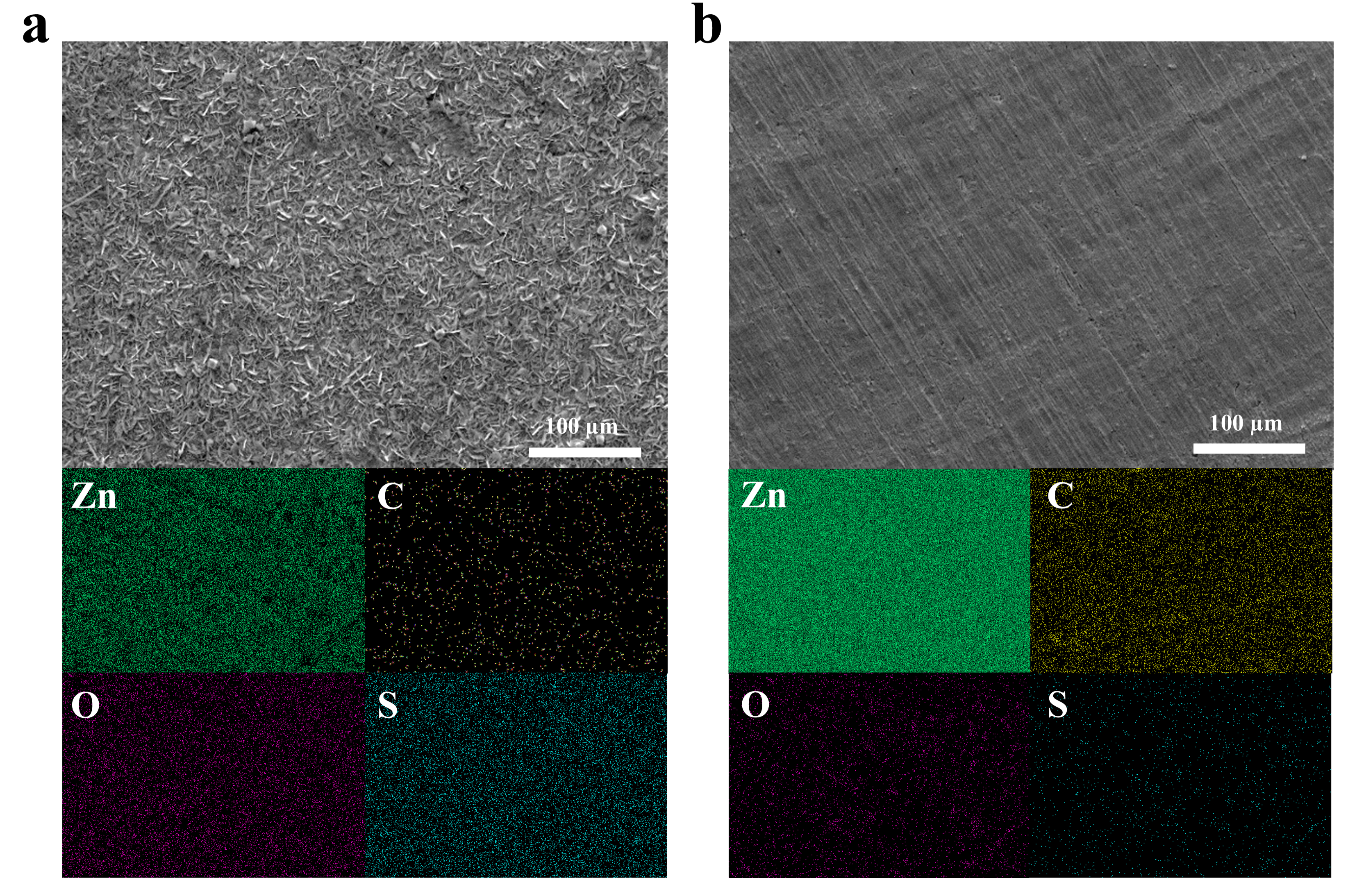


**Figure S11.** SEM images and EDS spectra of Zn foil immersed in (a) 2M ZnSO_4_ and (b) 2M ZnSO_4_ with OND for three days.





**Figure S12.** The Raman spectra of BE and OND/BE (300-500 cm^-1^).


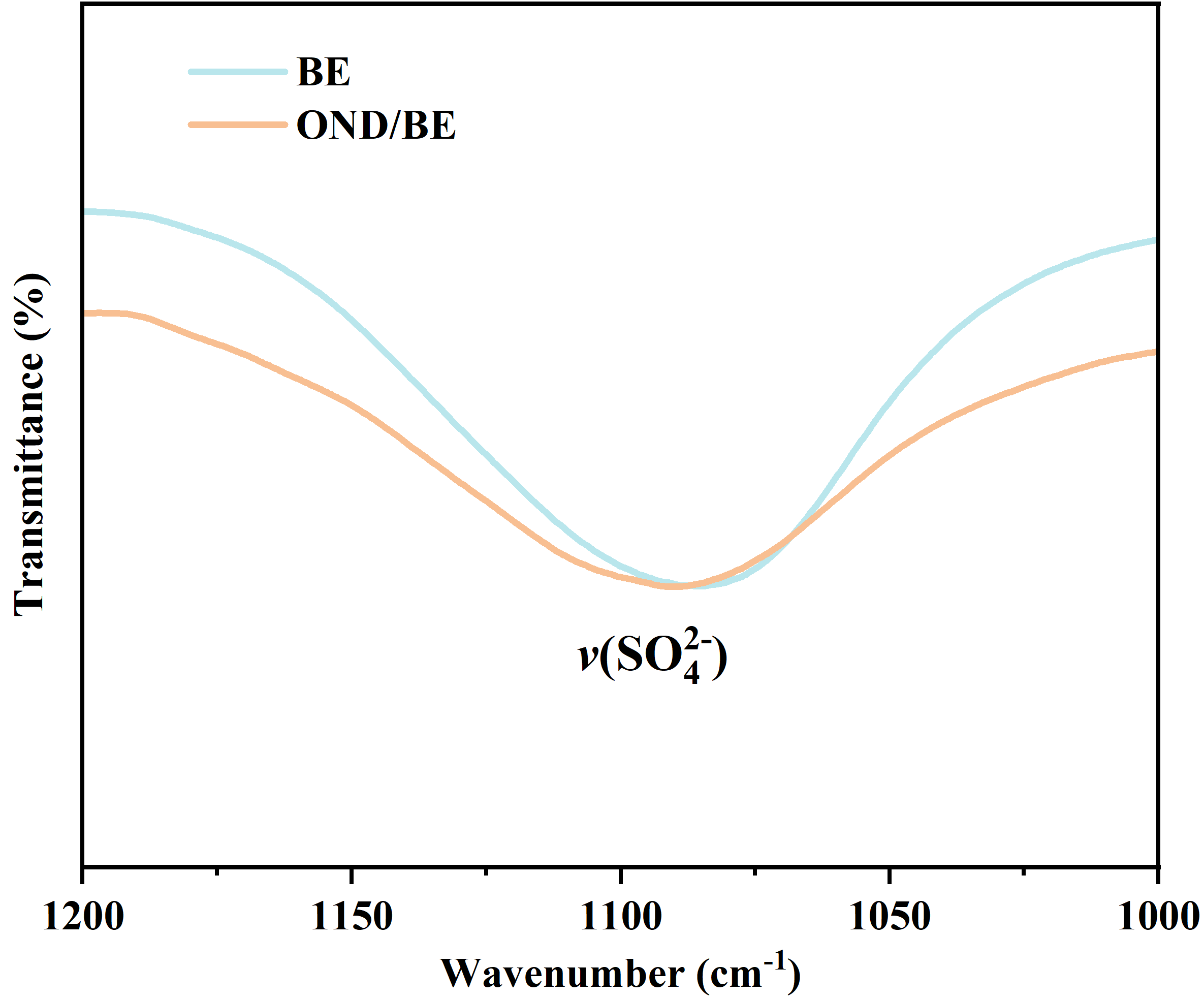


**Figure S13.** The infrared spectra of BE and OND/BE (1200-1000 cm^-1^).





**Figure S14.** Thermal conductivity of different electrolytes as a function of temperature.





**Figure S15.** Arrhenius plots and corresponding activation energies of Zn||Zn symmetric cells operated in electrolytes with and without OND additives at various temperatures.





**Figure S16.** The nucleation overpotential of Zn^2+^ in Zn//Cu batteries with different electrolytes.


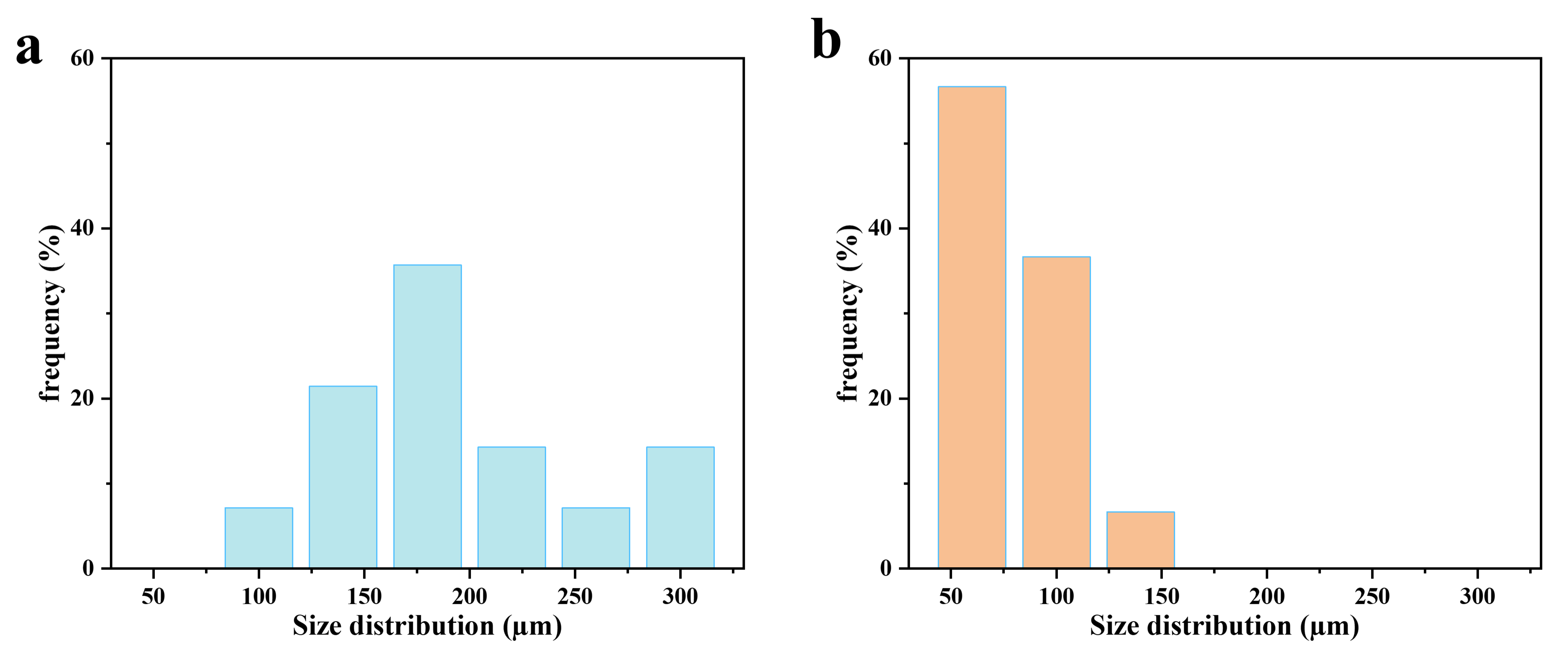


**Figure S17.** Using Cu as the positive electrode and Zn as the negative electrode, in two-electrode systems with different electrolytes, Zn^2+^ was continuously deposited on Cu foil at a current density of 1 mA cm^-2^ for 24 hours. Size distribution of Zn particles on the surface of Cu foil in (a) BE, (b) OND/BE.





**Figure S18.** Using the same zinc foil after 10, 25, and 50 cycles, the surface was scanned in the same region (2000 μm × 2000 μm). (a-f) CV curve of the probe before each SECM test. (g, h) Probe approach curve during the initial SECM test on the surface of Zn.


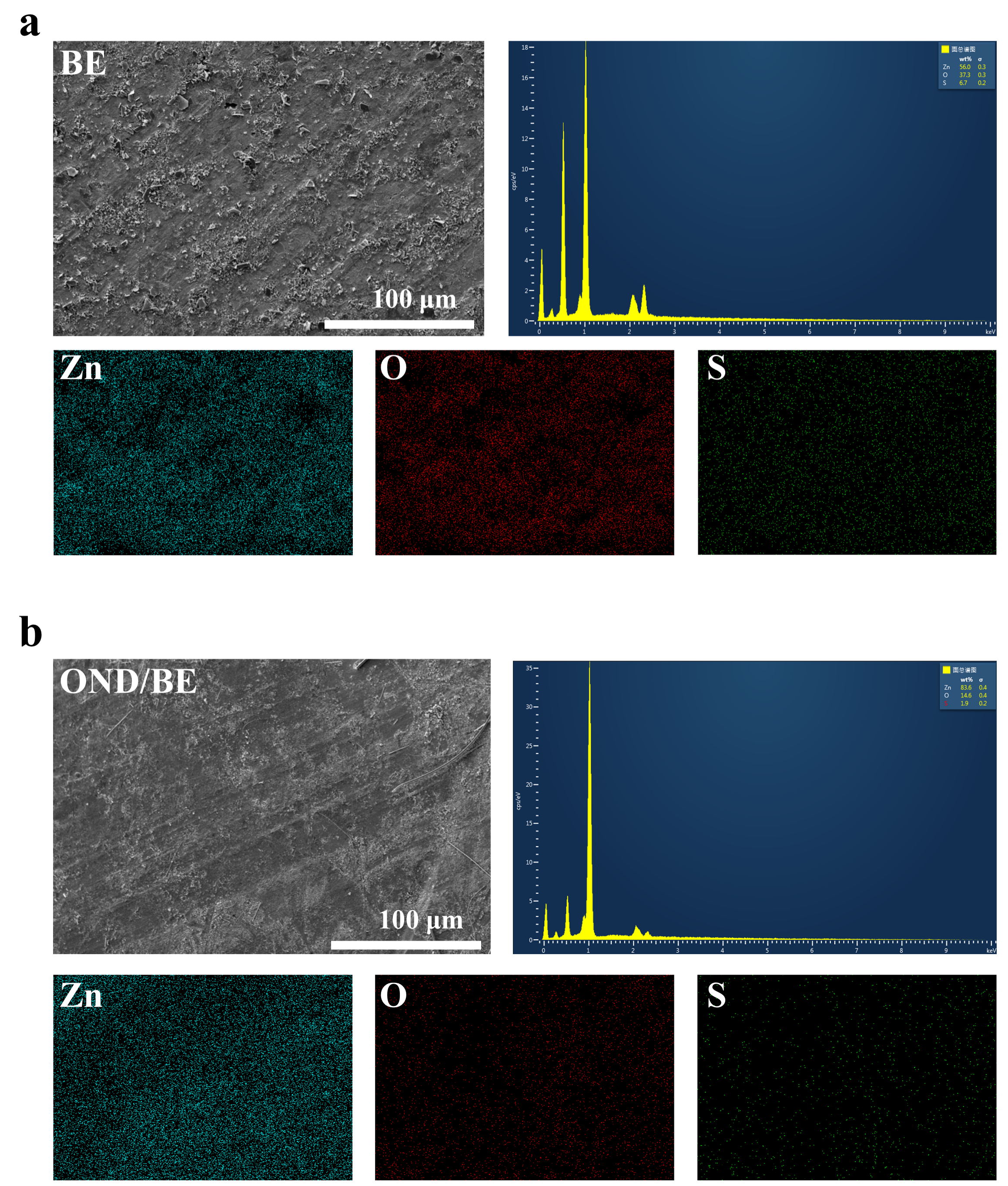


**Figure S19.** SEM images and corresponding EDS elemental mappings of Zn anodes after 50 cycles in (a) BE and (b) OND/BE at 1 mA cm^-2^ and 1 mAh cm^-2^.





**Figure S20.** Long-term galvanostatic Zn plating/stripping in Zn//Zn symmetric batteries in BE and OND/BE at 3 mA cm^-2^ with specific capacity of 1 mAh cm^-2^ (inset with magnified views of selected cycles of 400-404 h and 4000-4004 hours).


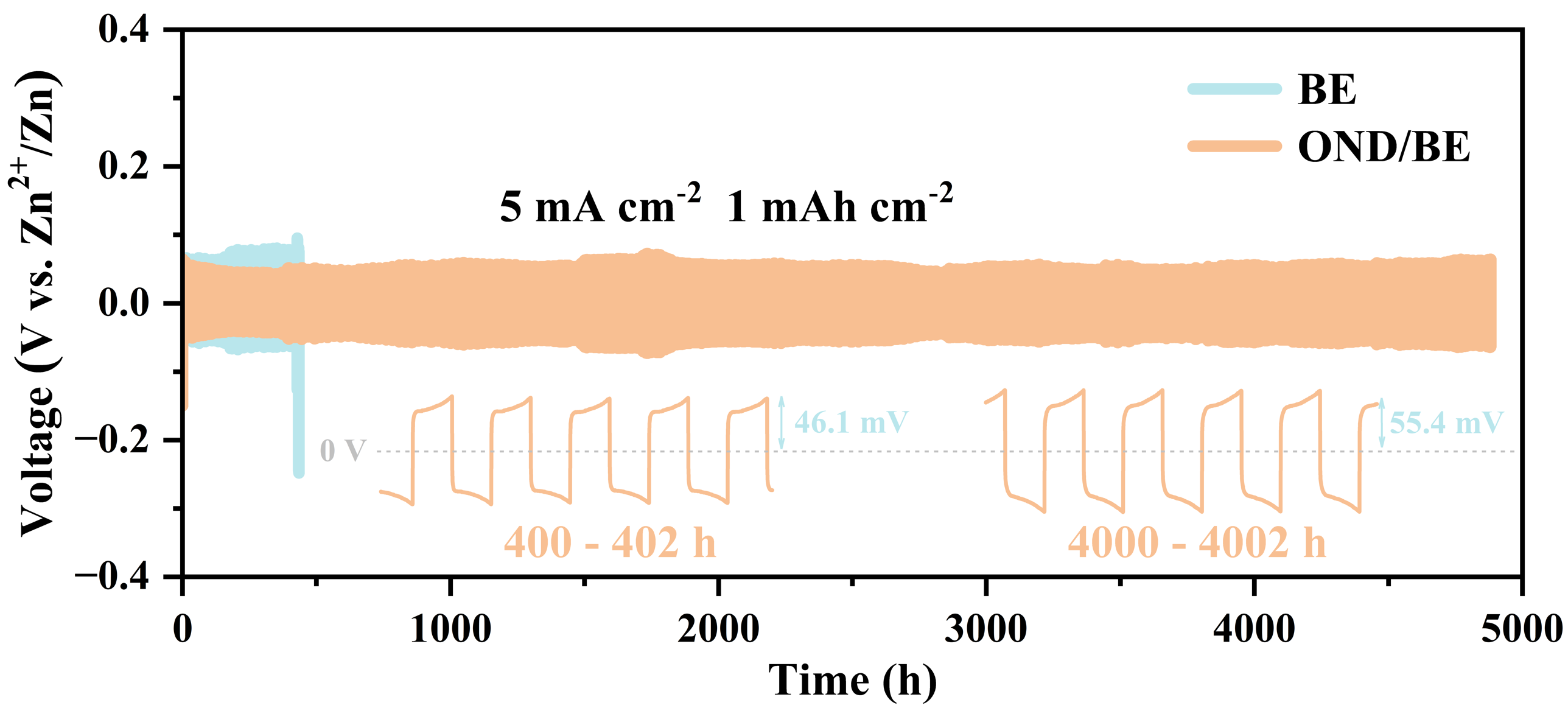


**Figure S21.** Long-term galvanostatic Zn plating/stripping in Zn//Zn symmetric batteries in BE and OND/BE at 5 mA cm^-2^ with specific capacity of 1 mAh cm^-2^ (inset with magnified views of selected cycles of 400-402 h and 4000-4002 hours).


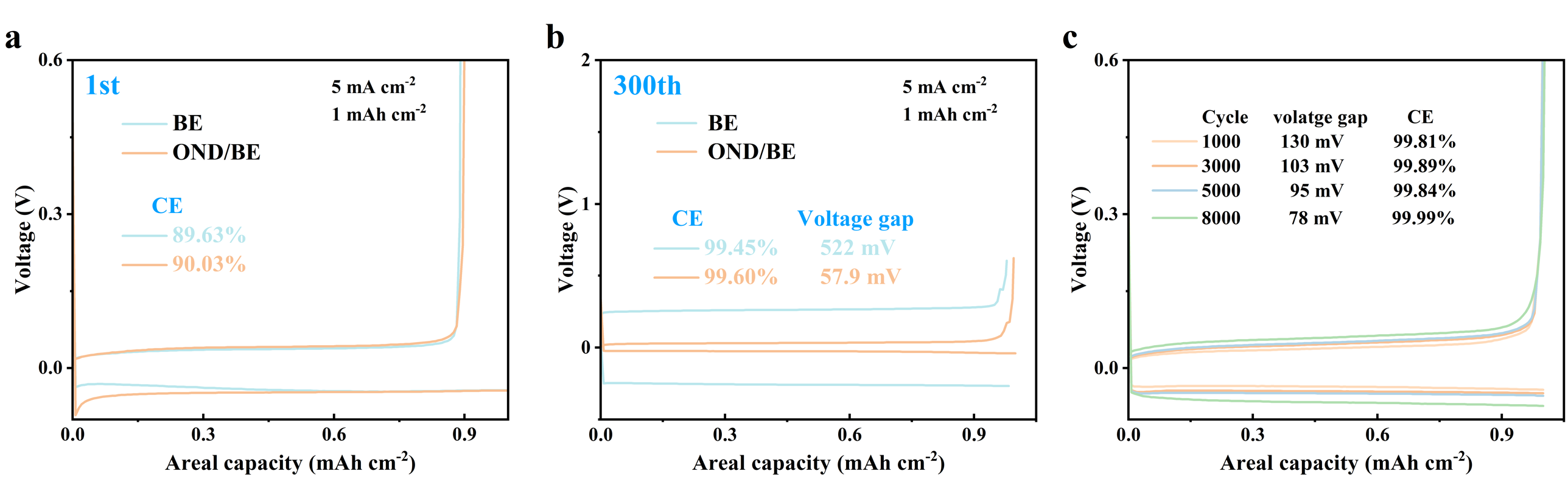


**Figure S22.** The voltage profiles of Zn//Cu half batteries in BE and OND/BE during the (a) 1st cycle, (b) 300th cycle. (c) The voltage profiles of Zn//Cu half batteries OND/BE during the 1000th, 3000th, 5000th and 8000th cycle.





**Figure S23.** CV curves of a Zn//Ti half-cell for the 1st, 2nd, 5th, and 10th cycles, along with the corresponding coulombic efficiency plots (a, b) in BE, (c, d) in OND/BE.


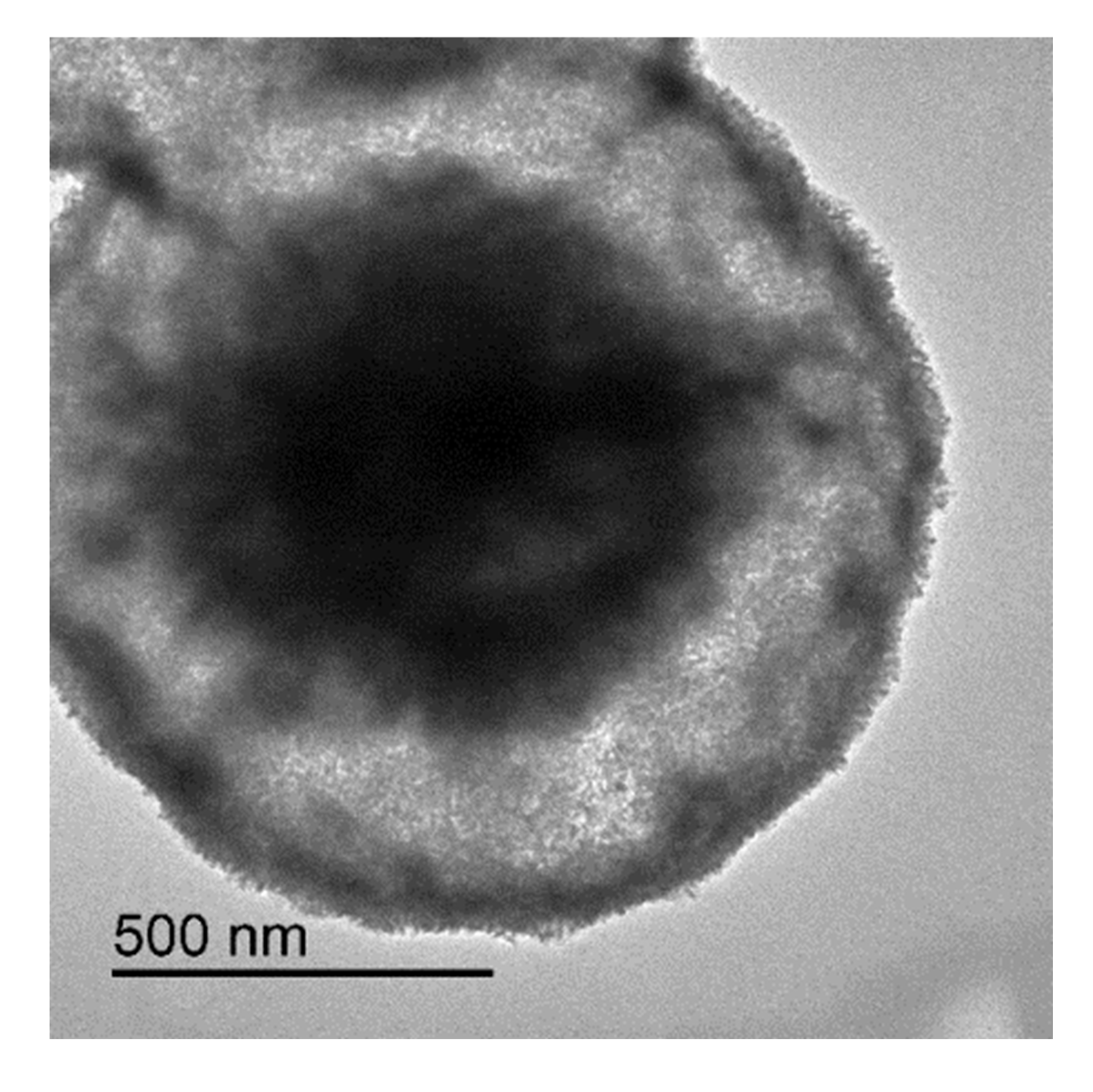


**Figure S24.** TEM image of MnO_2_.


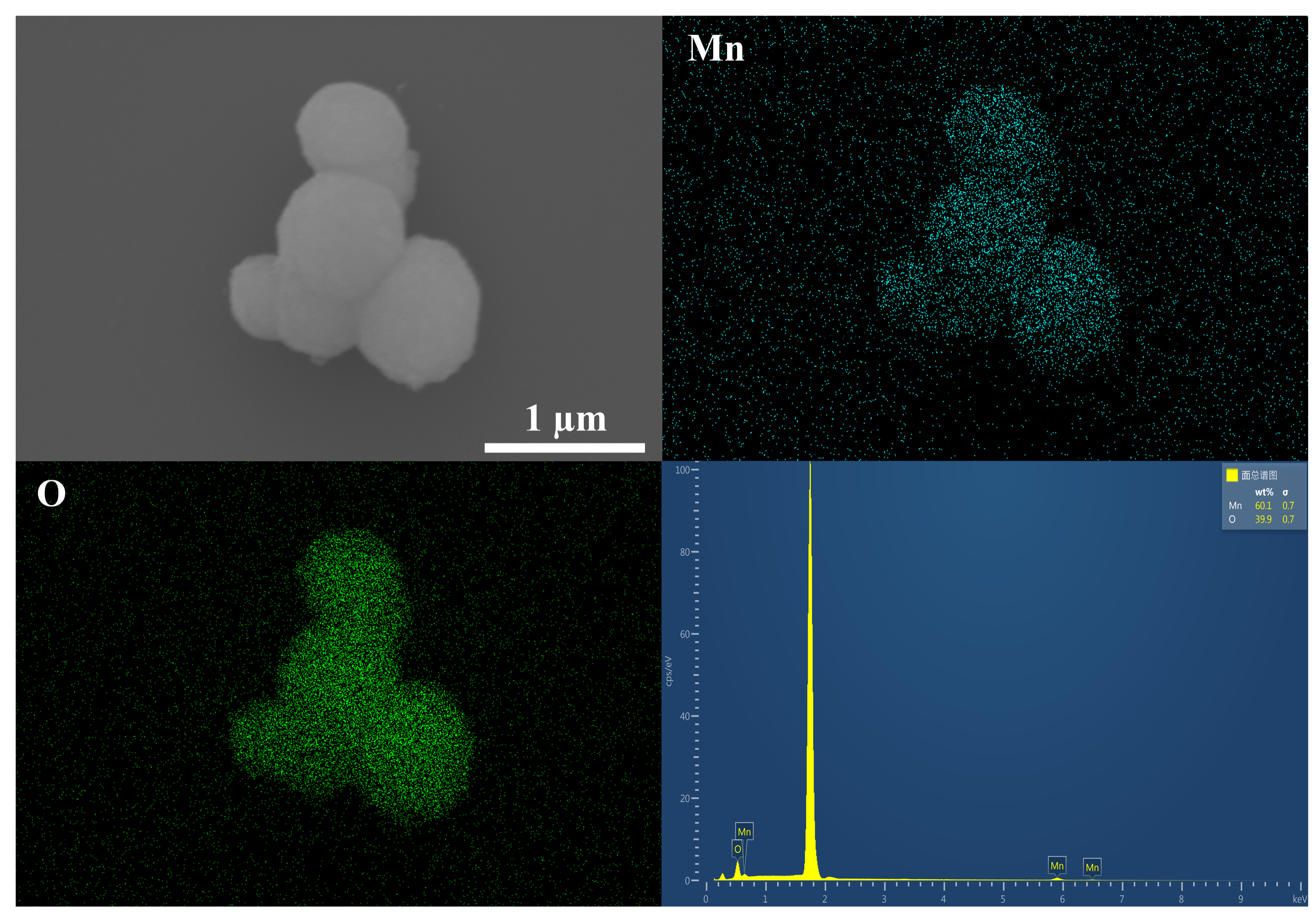


**Figure S25.** SEM image of MnO_2_ and corresponding EDS spectrum with elemental composition


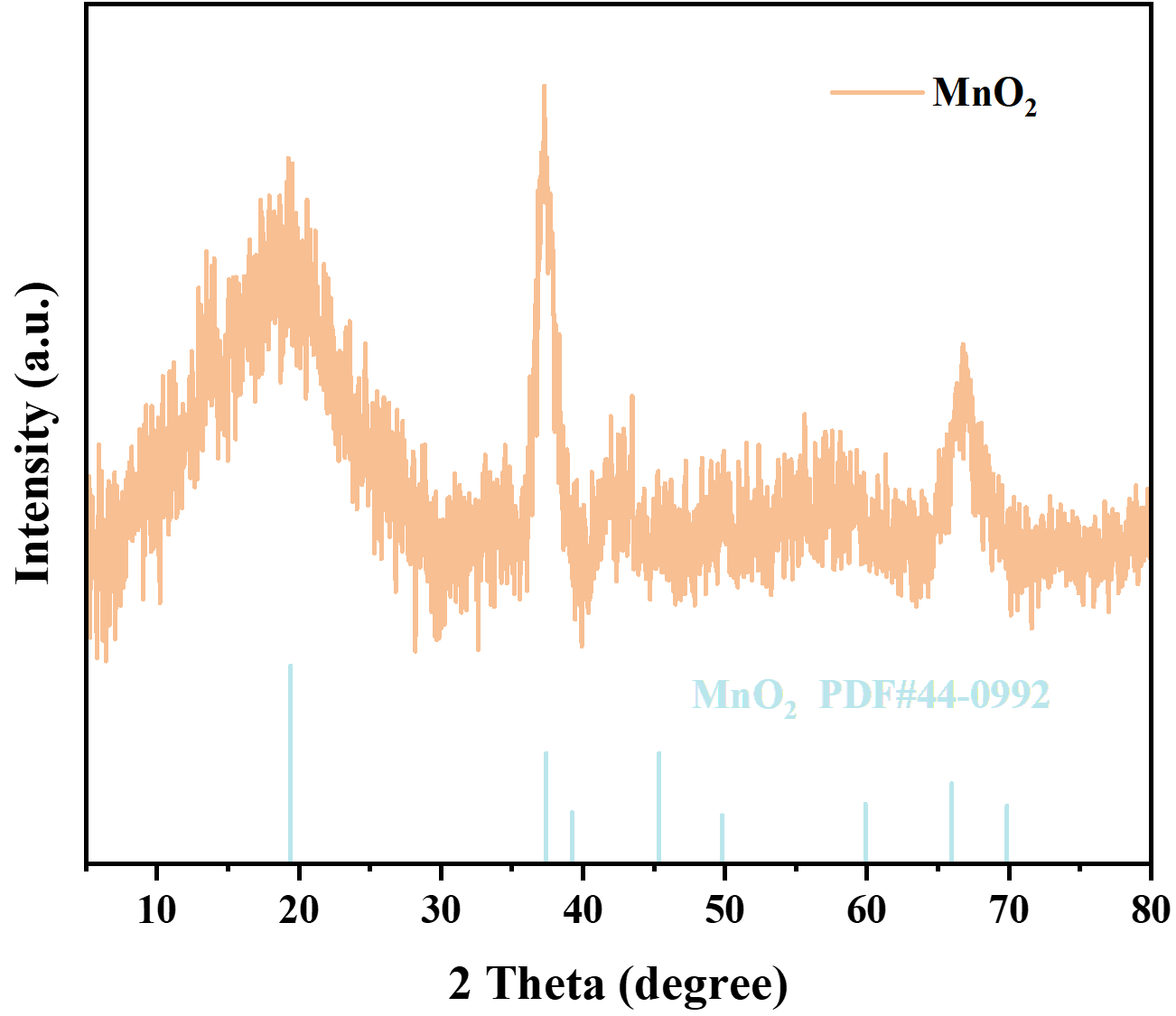


**Figure S26.** XRD pattern of MnO_2_.

**Table S1**. XPS results of ND and OND

| Sample | C (%) | O (%) |
| --- | --- | --- |
| ND | 89.3 | 8.7 |
| OND | 73.4 | 26.6 |

**Table S2**. The number of Zn^2+^ carried by a 300 nm OND

| Sample | c (Zn^2+^)  (mg L^-1^) | m (Zn^2+^)  (g) | N (Zn^2+^) | △N (Zn^2+^) | m (ND)  (g) | N (ND) | N (Zn^2+^):  N (ND) |
| --- | --- | --- | --- | --- | --- | --- | --- |
| BE | 102049 | 1.02049 | 9.4513*10^21 | 5.6495*10^18 | \ | \ | 280000 |
| OND/BE | 101988 | 1.01988 | 9.4456*10^21 |  | \ | \ |  |
| OND | \ | \ | \ | \ | 1 | 2.0096*10^13 |  |

**Table S3**. Comparison of the performance of OND with that of the high-performance AZIBs electrolyte additives/coating strategies reported in the literature for Zn//Zn batteries

| strategy | Current density (mA cm^-2^) | Cycle number | CPC  (mAh cm^-2^) | references |
| --- | --- | --- | --- | --- |
| amphiphilic polysorbate | 10 | 975 | 9750 | [4] |
| Dimethyl hydroxymethylphosphonate | 5 | 5612 | 11224 | [5] |
| 2,5-pyrroledione | 10 | 1050 | 1050 | [6] |
| polyacrylonitrile with fluorine-nitrogen co-doped carbon dots | 10 | 3100 | 3100 | [7] |
| zincophilic zinc tungstate | 10 | 1200 | 1200 | [8] |
| iron/zinc phosphate nanofilm | 10 | 14300 | 14300 | [9] |
| this work | 10 | 23000 | 23000 |  |

**Table S4**. Comparison of the performance of OND with that of the high-performance AZIBs electrolyte additives/coating strategies reported in the literature for Zn//MnO_2_ batteries

| strategy | Current density  (A g^-1^) | Cycle number | Decay rate per cycle (%) | references |
| --- | --- | --- | --- | --- |
| pyrocatechol violet | 1 | 1000 | 0.0363 | [10] |
| citric acid | 1 | 2000 | 0.0122 | [11] |
| disodium succinate | 2 | 2500 | 0.013 | [12] |
| protonated triglycine | 2 | 900 | 0.0372 | [13] |
| bis(2-hydroxyethyl) aminotris (hydroxymethyl) methane | 1 | 600 | 0.055 | [14] |
| cyclized polyacrylonitrile | 1 | 300 | 0.0333 | [15] |
| this work | 3 | 10000 | 0.0071 |  |

[1] Y. Fu, X. Gao, D. Zha, et al., "Yolk–shell-structured MnO2 microspheres with oxygen vacancies for high-performance supercapacitors," *J. Mater. Chem. A* **2018**, *6* (4), 1601, https://doi.org/10.1039/c7ta10058b.

[2] B. Hess, C. Kutzner, D. van der Spoel, E. Lindahl, "GROMACS 4: Algorithms for Highly Efficient, Load-Balanced, and Scalable Molecular Simulation," *J. Chem. Theory Comput.* **2008**, *4* (3), 435, https://doi.org/10.1021/ct700301q.

[3] L. Martinez, R. Andrade, E. G. Birgin, J. M. Martinez, "PACKMOL: a package for building initial configurations for molecular dynamics simulations," *J. Comput. Chem.* **2009**, *30* (13), 2157, https://doi.org/10.1002/jcc.21224.

[4] Z. Peng, S. Li, L. Tang, et al., "Water-shielding electric double layer and stable interphase engineering for durable aqueous zinc-ion batteries," *Nat Commun* **2025**, *16* (1), 4490, https://doi.org/10.1038/s41467-025-59830-y.

[5] H. Tan, C. Meng, Y. P. Zhang, et al., "Decoupling of Ion‐Solvent Interactions via Compartmentalized Molecular Design for Ultra‐Stable Aqueous Zinc Batteries," *Advanced Functional Materials* **2025**, https://doi.org/10.1002/adfm.202516270.

[6] Z. Dai, X. Zhang, C. Yang, et al., "Enhancing Zinc Anode Stability via Self‐Assembled Organic/Inorganic Hybrid Electrolyte Interfaces," *Advanced Energy Materials* **2025**, https://doi.org/10.1002/aenm.202503193.

[7] K. Wang, P. He, D. Xie, et al., "Modulating Zn2+ Desolvation and Deposition with Fluorine‐Nitrogen Co‐doped Carbon Dot Interlayers for High‐Rate Aqueous Zinc‐Ion Batteries," *Advanced Functional Materials* **2025**, https://doi.org/10.1002/adfm.202513796.

[8] J. Cao, H. Wu, D. Zhang, et al., "In-Situ Ultrafast Construction of Zinc Tungstate Interface Layer for Highly Reversible Zinc Anodes," *Angew Chem Int Ed Engl* **2024**, *63* (29), e202319661, https://doi.org/10.1002/anie.202319661.

[9] J. Li, J. Ba, C. Zhao, et al., "A Hydrophobic and High Surface Charge Phosphate Interphase for High Areal Capacity Zinc Metal Batteries," *Adv Mater* **2025**, *37* (34), e2501956, https://doi.org/10.1002/adma.202501956.

[10] W. Liang, D. Li, R. Zhong, et al., "Electrolyte Engineering Strategy with Catecholate Type Additive Enabled Ultradurable Zn Anode," *Advanced Functional Materials* **2025**, *35* (36), https://doi.org/10.1002/adfm.202504195.

[11] T. Xue, Y. Mu, Z. Zhang, et al., "Enhanced Zinc Deposition and Dendrite Suppression in Aqueous Zinc‐Ion Batteries Via Citric Acid‐Aspartame Electrolyte Additives," *Advanced Energy Materials* **2025**, *15* (26), https://doi.org/10.1002/aenm.202500674.

[112] Y. Ding, L. Yin, T. Du, et al., "A Trifunctional Electrolyte Enables Aqueous Zinc Ion Batteries with Long Cycling Performance," *Advanced Functional Materials* **2024**, *34* (30), https://doi.org/10.1002/adfm.202314388.

[13] J. Zhang, Y. Liu, Y. Wang, Z. Zhu, Z. Yang, "Zwitterionic Organic Multifunctional Additive Stabilizes Electrodes for Reversible Aqueous Zn‐Ion Batteries," *Advanced Functional Materials* **2024**, *34* (34), https://doi.org/10.1002/adfm.202401889.

[14] Y. M. Li, W. H. Li, X. Y. Zhang, et al., "Multifunctional pH‐Controlling Electrolyte Enables Ultrastable and Highly Reversible Zinc Anode," *Advanced Functional Materials* **2024**, *35* (15), https://doi.org/10.1002/adfm.202420446.

[15] J. Yang, S. Wang, L. Du, et al., "Thermal‐Cyclized Polyacrylonitrile Artificial Protective Layers Toward Stable Zinc Anodes for Aqueous Zinc‐Based Batteries," *Advanced Functional Materials* **2024**, *34* (21), https://doi.org/10.1002/adfm.202314426.
